# CDK13 upregulation-induced formation of the positive feedback loop among circCDK13, miR-212-5p/miR-449a and E2F5 contributes to prostate carcinogenesis

**DOI:** 10.1186/s13046-020-01814-5

**Published:** 2021-01-04

**Authors:** Jin-Chun Qi, Zhan Yang, Tao Lin, Long Ma, Ya-Xuan Wang, Yong Zhang, Chun-Cheng Gao, Kai-Long Liu, Wei Li, An-Ning Zhao, Bei Shi, Hong Zhang, Dan-Dan Wang, Xiao-Lu Wang, Jin-Kun Wen, Chang-Bao Qu

**Affiliations:** 1 Department of Urology, The Second Hospital of Hebei Medical University, 215 Heping W Rd, Shijiazhuang, 050000 China; 2 Department of Biochemistry and Molecular Biology, Ministry of Education of China, Hebei Medical University, No. 361 Zhongshan E Rd, Shijiazhuang, 050017 China

**Keywords:** Prostate cancer, CDK13, circRNAs biogenesis, E2F5, Drug resistance

## Abstract

**Background:**

Both E2F transcription factor and cyclin-dependent kinases (CDKs), which increase or decrease E2F activity by phosphorylating E2F or its partner, are involved in the control of cell proliferation, and some circRNAs and miRNAs regulate the expression of E2F and CDKs. However, little is known about whether dysregulation among E2Fs, CDKs, circRNAs and miRNAs occurs in human PCa.

**Methods:**

The expression levels of CDK13 in PCa tissues and different cell lines were determined by quantitative real-time PCR and Western blot analysis. In vitro and in vivo assays were preformed to explore the biological effects of CDK13 in PCa cells. Co-immunoprecipitation anlysis coupled with mass spectrometry was used to identify E2F5 interaction with CDK13. A CRISPR-Cas9 complex was used to activate endogenous CDK13 and circCDK13 expression. Furthermore, the mechanism of circCDK13 was investigated by using loss-of-function and gain-of-function assays in vitro and in vivo.

**Results:**

Here we show that CDK13 is significantly upregulated in human PCa tissues. CDK13 depletion and overexpression in PCa cells decrease and increase, respectively, cell proliferation, and the pro-proliferation effect of CDK13 is strengthened by its interaction with E2F5. Mechanistically, transcriptional activation of endogenous CDK13, but not the forced expression of CDK13 by its expression vector, remarkably promotes E2F5 protein expression by facilitating circCDK13 formation. Further, the upregulation of E2F5 enhances CDK13 transcription and promotes circCDK13 biogenesis, which in turn sponges miR-212-5p/449a and thus relieves their repression of the E2F5 expression, subsequently leading to the upregulation of E2F5 expression and PCa cell proliferation.

**Conclusions:**

These findings suggest that CDK13 upregulation-induced formation of the positive feedback loop among circCDK13, miR-212-5p/miR-449a and E2F5 is responsible for PCa development. Targeting this newly identified regulatory axis may provide therapeutic benefit against PCa progression and drug resistance.

## Background

Prostate cancer (PCa) is one of the most common malignancies and the second leading cause of cancer-related death in men [1, 2]. Although organ-confined PCa can be effectively treated by radical prostatectomy or radiation therapies, androgen deprivation therapy (ADT) is first-line *treatment* for metastatic PCa. Once hormonal resistance occurs, PCa progresses rapidly, and advanced PCa is usually fatal within 18 months [3, 4]. Currently, several compounds, including abiraterone acetate [5], enzalutamide [6], sipuleucel-T [7], alpharadin [8], and docetaxel [9] have been used to help treat PCa. Unfortunately, adverse side effects of the treatment and drug resistance often lead to treatment failure [10]. Therefore, there is an urgent need to further understand the molecular mechanism involved in prostate carcinogenesis and drug resistance.

Although the molecular mechanisms driving prostate carcinogenesis are complex, the dysregulation of cell proliferation is a fundamental feature of all types of cancer. Cell proliferation is coupled with cell cycle progression, and mammalian CDKs are essential for driving each cell cycle phase. Accumulating evidence has suggested that tumor-associated cell cycle disorders are often mediated by alterations in cyclin-dependent kinase (CDK) activity. Mis-regulated CDKs induce unscheduled proliferation [11]. It has been well known that mammalian cells contain at least 13 CDKs [11]. Of these, CDK1-CDK6, CDK10 and CDK11 are all involved in cell cycle control [11–13]. CDK7,CDK8 and CDK9 have activities that are different from cell cycle control, these 3 CDKs can phosphorylate the carboxyl-terminal domain (CTD) of RNA polymerase II and exert actions in transcriptional regulation [12–14]. CDK12 and CDK13 bind to L-type cyclins (CycL) and regulate alternative RNA splicing [15, 16]. A recent study shows that knocking out CDK13 leads to abnormal expression of several genes involved in a variety of biological processes including cell growth regulation [17]. Notably, both CDK12 and CDK13 knockdown affect the expression of genes involved in RNA processing, but CDK13-regulated gene sets are not affected by CDK12 knockdown. These evidences clearly suggest that human CDK functions do not overlap with each other, probably reflecting tissue-specific and fine-tuned regulation of cell cycle regulation. Importantly, several recent studies reported that CDK12 expression is dysregulated in metastatic castration-resistant prostate cancer (mCRPC) samples, and CDK12 loss results in highly recurrent gains at loci of genes involved in the cell cycle and DNA replication [18–20]. However, much less is known regarding CDK13 expression and function in PCa.

Circular RNAs (circRNAs) are a novel class of non-coding RNA characterized by the presence of a covalent bond linking the 3′ and 5′ ends generated by back-splicing [21]. Emerging evidences have shown that circRNAs are frequently deregulated in various diseases and have distinct and specific functions in a number of biological processes, such as proliferation, apoptosis or drug resistance [22, 23]. Our previous study revealed that the RNA-binding protein RBM25 induces circAMOTL1L biogenesis by directly interacting with circAMOTL1L, p53 upregulates circAMOTL1L expression through activating the RBM25 gene, whereas p53 downregulation in PCa cells facilitates epithelial-mesenchymal transition (EMT) [24]. Recently, we found that circACTA2 is able to mediate NRG-1-ICD regulation of its parental gene ACTA2 (alpha-actin gene) in vascular smooth muscle cells via NRG-1-ICD/circACTA2/miR-548f-5p axis [25]. Remarkably, several lines of evidence suggest that some circRNAs play important roles in the resistance of cancer cells to anticancer drugs. For example, circAKT3 upregulates PIK3R1 to enhance cisplatin resistance in gastric cancer via inhibition of miR-198 [22]. circ_0025202 suppresses tumor growth and enhances tamoxifen sensitization via regulating the miR-182-5p/FOXO3a axis in breast cancer [26]. Circular RNA cESRP1 increases small cell lung cancer responsiveness to chemotherapy by sequestering miR-93-5p to inhibit the TGF-β pathway [27]. So far, the multiple mechanisms that have been reported to be associated with the development of drug resistance involve alterations in drug targets, drug metabolism, cancer stem cell population, DNA damage repair, as well as cell survival and death signals [28]. Despite the important roles of circRNAs in prostate carcinogenesis and drug resistance, little is known about the role of circRNAs derived from the same parental gene, which can regulate the transcription of the parental gene by binding to RNA polymerase II [29], and thus form the positive feedback loop between circRNAs and their parental genes to induce prostate carcinogenesis and drug resistance.

In this study, we report that CDK13 is significantly upregulated in PCa, and transcriptional activation of endogenous CDK13 promotes E2F5 expression by facilitating the formation of circCDK13, which in turn sponges miR-212-5p/449a and thus relieves their repression of the E2F5 expression, subsequently leading to the upregulation of E2F5 expression. Further, increased E2F5 enhances CDK13 transcription and promotes circCDK13 biogenesis. Our findings provide the first evidence that CDK13 upregulation-induced formation of the positive feedback loop among circCDK13, miR-212-5p/miR-449a and E2F5 contributes to prostate carcinogenesis and drug resistance.

## Methods

The detailed procedures of plasmid and lentivirus expression vector constructs, antibody and immunoblot, xenograft animal model, RNA isolation and RT-qPCR, cell proliferation assays, chromatin immunoprecipitation-qPCR, co-immunoprecipitation assay, immunofluorescence staining, in situ hybridization, morphometry and histology, luciferase reporter assay, analyses of apoptosis, RNA synthesis and biotin pull-down, TUNEL staining, RNA immunoprecipitation (RIP) assays, proximity ligation assay as well as key reagents are described in Supplementary Experimental Procedures.

### Clinical samples and microarray

Clinical samples collection and clinicopathological characteristics as described previously [24, 30]. In brief, Patients underwent radical prostatectomy for localized PCa and benign prostatic hyperplasia underwent transurethral resection of the prostate (TURP) at the Department of Urology, the Second Hospital of Hebei Medical University, China from July 2014 to October 2017. No treatment was administered prior to surgery. All the tissue specimens were confirmed by two experienced pathologists. The study protocol was approved by the Ethics Committee of Second Hospital of Hebei Medical University and Verbal consent was obtained from each patient. Microarray hybridization analysis of mRNA expression in 2 PCa samples and 2 BPH were performed according to the manufacturer’s protocol (Arraystar, Inc., Rockville, MD, USA).

### Cell culture and transfections

PC3 (CRL-1435; ATCC), LNCaP (CRL-1740; ATCC), 22Rv1 (CRL-2505; ATCC) and DU145 cells (HTB-81; ATCC) were grown and maintained in RPMI 1640 medium (Gibco, USA) containing penicillin (100 units/ml) and streptomycin (100 μg/ml). RWPE-1 cells (CRL-11609; ATCC) were maintained in K-SMF medium (Life Technologies, USA) supplemented with 5 ng/mL epidermal growth factor (EGF) and 50 μg/mL bovine pituitary extract. Cell Cultures and transfections were performed as described previously [24, 30]. In brief, the transfection was using Lipofectamine 2000 (Invitrogen) according to the manufacturer’s protocols. The miR-449a and miR-212-5p mimics, mimic NC, inhibitors, inhibitor NC, circRNA probe and its controls were purchased from GenePharma Co., Ltd. (Shanghai, China).

### Activation of endogenous genes by a CRISPR-Cas9 complex

In order to activate endogenous CDK13 we used a CRISPR-Cas9 complex with three SAM components: dCas9–VP64 (Addgene #61425),MS2–p65–HSF1(Addgene, #61426), and sgRNA (Addgene, #89493) as described previously [31]. gRNAs were designed using the online optimized CRISPR design tool (http://crispr.mit.edu) and targeted the proximal promoter regions of CDK13. Oligos, synthesized by Sangon Biotech., (Shanghai, China), were annealed and sub-cloned into the lentiGuide-puro vector.

### Statistical analysis

All of the data are presented as the means±SEM. Student’s t-test was used to analysis the differences between two groups for multiple comparisons or repeated measurements, ANOVA or repeated ANOVA followed by Tukey’s post hoc test was used. *P* < 0.05 was considered statistically significant. Statistical analysis was performed using Graphpad Prism 7 software (GraphPad Software, San Diego, CA, USA).

## Results

### CDK13 is upregulated in PCa tissues

**Fig. 1:**
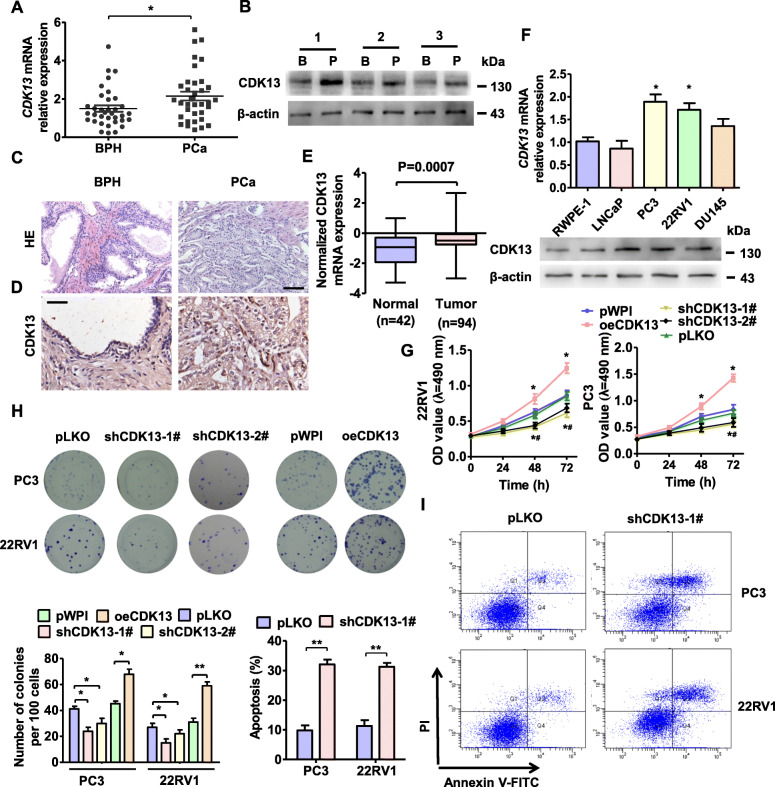
CDK13 is upregulated in PCa tissues and the upregulation of CDK13 promoted proliferation in PCa cells. **a**, RT-qPCR detected CDK13 mRNA expression in 30 pairs of PCa and benign prostatic hyperplasia (BPH). *P < 0.05 vs. BPH. **b**, CDK13 protein expression was measured by Western blotting in 3 pairs of randomly selected BPH (B) and PCa (P) tissues. **c**, Hematoxylin and eosin (HE) staining of BPH and PCa tissues. **d**, Immunochemistry staining of CDK13 in BPH and PCa tissues. Scale bar = 50 mm. **e**, The expression levels of CDK13 mRNA in BPH and PCa tissues from the GSE13507 database (*P* = 0.0007). **f**, The expression of CDK13 was examined by RT-qPCR (up panel) or Western blotting (down panel) in human normal prostate epithelial cells (RWPE-1) and PCa cell lines (LNCaP, PC3, 22RV1 and DU145). CDK13 expression was significantly increased in PC3 and 22RV1 cell lines. *P < 0.05 vs. RWPE-1. **g** and **h**, Cell viability was measured by MTS assay (G) and colony formation assays (H) in PC3 and 22RV1 cell lines after transfected with indicated vectors. *P < 0.05, and **P < 0.01 vs. their respective empty vector. **i**, PC3 and 22RV1 cells were transfected with shCDK13 or control vector, cell apoptosis was detected by AnnexinV/PI flow cytometry. Right panel shows the apoptosis rate of three independent experiments. **P < 0.001 vs. pLKO. pWPI is a control vector of oeCDK13 and pLKO is the control vector of shCDK13

To evaluate the potential role of CDK13 in the development of human PCa, we first examined the CDK13 mRNA expression in 30 PCa tissues and benign prostatic hyperplasia (BPH) by using RT-qPCR. As shown in Fig. 1a, CDK13 mRNA level was significantly increased in PCa tissues compared with BPH tissues. Western blot analysis also confirmed that PCa tissues had higher levels of CDK13 protein than BPH tissues (Fig. 1b, Appendix Fig. S1A). Consistently, a significantly increased expression level of CDK13 was observed in the PCa tissues by the immunohistochemistry staining (Fig. 1c, d and Appendix Fig. S1B). Furthermore, we analyzed the CDK13 expression level in the TCGA database and found that the CDK13 mRNA levels were significantly increased in PCa tissues (*n* = 94) compared with normal prostate tissues (*n* = 42) (Fig. 1e). These findings suggest that CDK13 upregulation is correlated with the development of human PCa.

### Upregulation of CDK13 promotes proliferation and inhibits apoptosis in PCa cells in vitro

Next, we examined CDK13 expression in four different PCa cell-lines (LNCaP, PC3, 22RV1 and DU145) by RT-qPCR and Western blot analysis. The results showed that protein and mRNA levels of CDK13 were significantly upregulated in PC3 and 22RV1 cells compared with normal prostatic epithelium cell line RWPE-1 cells, consistent with the observations in PCa tissues (Fig. 1f, Appendix Fig. S1C). Thus, PC3 and 22RV1 cells were chosen in all subsequent experiments. To determine whether the upregulation of CDK13 is responsible for PCa development, we overexpressed or knocked down CDK13 in these two cell lines by transfection of CDK13 expression plasmids or plasmids expressing CDK13 short hairpin RNA and detected CDK13 expression (Appendix Fig. S1D). Then we performed loss- and gain-of-function experiments to investigate the functions of CDK13 in proliferation and apoptosis of PCa cells. As a result, overexpression of CDK13 in PC3, 22RV1 and RWPE-1 cells significantly promoted cell proliferation, whereas silencing of CDK13 inhibited PCa cell proliferation, as shown by the MTS assay (Fig. 1g, Appendix Fig. S1E). This result was further supported by colony formation assay, showing that the number of clones obviously increased or decreased upon CDK13 overexpression or silencing, respectively (Fig. 1h). Moreover, knockdown of CDK13 in PC3 and 22RV1 cells by shCDK13 significantly reduced cell apoptosis, as evidenced by flow cytometry of Annexin V-FITC/PI staining (Fig. 1i). Taking together, these data indicate that CDK13 plays an essential role in the regulation of PCa cell proliferation and apoptosis.

### CDK13 interacts with E2F5 which is upregulated in PCa

**Fig. 2:**
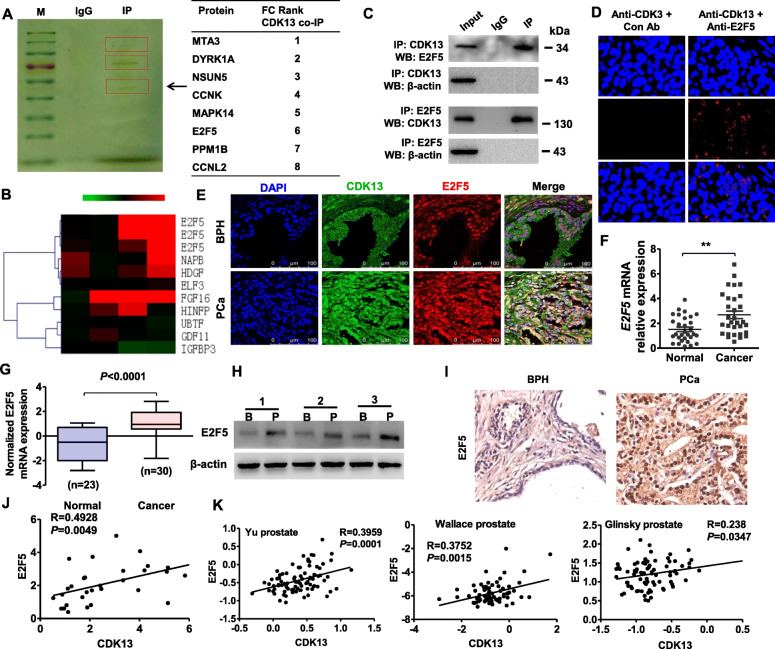
CDK13 interacts with E2F5 in PCa cells. **a**, Co-immunoprecipitation coupled with mass spectrometry (CoIP-MS) was performed with an anti-CDK13 in PC3 cells to detect the proteins interacting with CDK13, which are shown on the right panel. **b**, Heat map showing the differential expression (fold changes) of mRNAs between PCa and BPH tissues. Red color indicates several genes that are known to be transcriptionally upregulated in PCa tissues. **c**, CoIP analysis was used to detect the interaction between CDK13 and E2F5, and β-actin was used as a negative control. **d**, in situ proximity ligation (PLA) analysis detected the interaction between CDK13 and E2F5. Red color indicates PLA-positive cells. **e**, Immunofluorescence staining was performed to detect the expression and location of CDK13 and E2F5 in PCa and BPH tissues. Scale bars = 100 μm. **f**, E2F5 mRNA level was determined by RT-qPCR in 30 pairs of PCa and normal prostate tissues. **P < 0.01 vs. normal tissues. **g**, The expression levels of E2F5 mRNA in BPH and PCa tissues from the GSE13507 database (*P* < 0.0001). **h**, E2F5 protein expression was detected by Western blotting in 3 pairs of randomly selected BPH (B) and PCa (P) tissues. **i**, Immunohistochemistry staining detected the E2F5 protein expression in PCa and BPH tissues. **j** and **k**, the correlation between CDK13 and E2F5 mRNA expression in the PCa tissues was analyzed by Pearson correlation analysis in our clinical data (*R =* 0.4928, *P* = 0.0049) or several other data published in TCGA database (Yu: *R =* 0.3959, *P* = 0.0001; Wallace: *R =* 0.3752, *P* = 0.0015; Glinsky: *R =* 0.238, *P* = 0.0347)

Because most CDKs function by interacting with their partner proteins [32], we sought to know CDK13’s partner in the PCa cells. To do this, we performed co-immunoprecipitation coupled with mass spectrometry (CoIP-MS) and found that 25 proteins, including transcription factor E2F5, might interact with CDK13 (Fig. 2a). Besides, we detected the mRNA expression in 2 pairs of PCa and BPH tissues by using microarray and found that the expression level of E2F5 was markedly increased in the PCa tissues (Fig. 2b). The results of the above two experiments prompted us to investigate whether E2F5 interacts with CDK13 and participates in the development of PCa. As expected, reciprocal immunoprecipitation with anti-CDK13 or anti-E2F5 showed that there existed a strong interaction between endogenous CDK13 and E2F5 in the PC3 cells (Fig. 2c). A similar result was obtained by in situ proximity ligation (PLA) assay, showing a direct binding between CDK13 and E2F5 (Fig. 2d). Moreover, immunofluorescence staining showed that endogenous CDK13 and E2F5 were co-localized in the PCa tissues (Fig. 2e). These results clearly indicate that CDK13 interacts with E2F5 in PC3 cells and PCa tissues.

Next, we used RT-qPCR to examine the expression of E2F5 mRNA in human PCa tissues and found that the expression level of E2F5 mRNA was significantly higher in the PCa tissues (*n* = 30) than in normal prostate tissues (*n =* 30) (Fig. 2f). To provide additional confirmation, we analyzed E2F5 expression level in the TCGA database. Consistent with our results, E2F5 expression was substantially upregulated in the PCa tissues (*n =* 30) relative to normal prostate tissues (*n* = 23) (Fig. 2g). In parallel with alterations in mRNA, Western blot analysis and immunohistochemistry staining also revealed that PCa tissues had a higher protein level of E2F5 (Fig. 2h and i, Appendix Fig. S2A and B). To provide additional confirmation that CDK13 and E2F5 expression are differentially expressed in human PCa, prostatectomy specimens of patients with high-grade PCa (Gleason> 8), low-grade PCa (Gleason< 6) and benign prostatic hyperplasia (BPH) were examined by qRT-PCR and western blot. The result showed that CDK13 and E2F5 protein and mRNA expression were significantly upregulated in high-grade PCa tissues compared with low-grade PCa or BPH tissues (Appendix Fig. S2 C and D). Further, the correlation between E2F5 mRNA and CDK13 mRNA expression in the PCa tissues was analyzed, and there is a significant positive correlation between the two RNA levels (Fig. 2j). This positive correlation was further confirmed by the analysis of TCGA database (Fig. 2k). Collectively, these data suggest that E2F5 is upregulated in PCa and associates with CDK13.

### CDK13 and E2F5 cooperatively promote PCa cell proliferation by interacting with each other

**Fig. 3:**
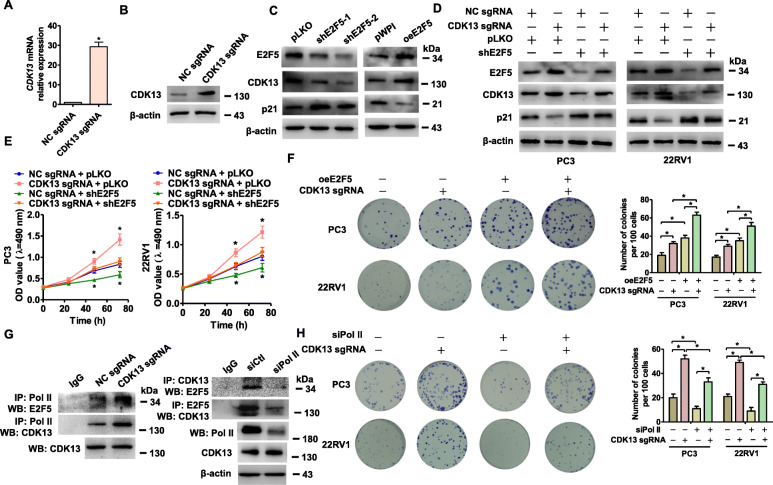
CDK13 interacts with E2F5 to cooperatively promote PCa cell proliferation. **a** and **b**, RT-qPCR and Western blot analysis detected the CDK13 expression in PC3 cells co-transfected with dCas9–VP64, MS2–p65–HSF1 and CDK13 promoter sgRNA (hereafter abbreviated as CDK13 sgRNA) or negative control (NC) sgRNA. *P < 0.05 vs NC sgRNA. **c**, Western blot analysis detected the E2F5, CDK13 and p21 expression in PC3 cells transfected with shE2F5, oeE2F5 or their respective control vector. **d**, Western blot analysis detected the proteins described as in (C) in PC3 and 22RV1 cells transfected with CDK13 sgRNA, shE2F5 or their respective control vector. **e**, Cells were prepared as in (D), cell viability was measured by MTS assay. *P < 0.05 vs. their respective controls. **f**, PC3 and 22RV1 cells were transfected with oeE2F5 and CDK13 sgRNA either alone or together, and colony formation assay was performed. Right panel shows the quantitative analysis of colony numbers from three independent experiments. *P < 0.05 vs. control constructs or CDK13 sgRNA and oeE2F5 alone. **g**, PC3 cells were transfected with CDK13 sgRNA or negative control (NC) sgRNA (left panel), as well as with siPol ll or siCtl (right panel). CoIP analysis detected the interaction between E2F5, CDK13 and Pol ll. **h**, Colony formation assay detected the proliferation of PC3 and 22RV1 cells co-transfected with the indicated constructs. Right panel shows the quantitative analysis of colony numbers from three independent experiments. *P < 0.05 vs. control constructs or siPol ll and CDK13 sgRNA alone

To further clarify the roles of CDK13 and E2F5 in PCa cell proliferation, we used a CRISPR-Cas9 complex to activate the endogenous CDK13 transcription [31]. As shown in Fig. 3a and b, transfection of sgRNA plasmid with CDK-specific targeting sequence (CDK13 sgRNA) could significantly increase the expression of CDK13 mRNA and protein compared with control sgRNA (Appendix Fig. S3A). Notably, knockdown of E2F5 in PC3 cells by transfecting plasmids expressing E2F5 short hairpin RNA (shE2F5) remarkably decreased the expression level of E2F5 and CDK13 and increased the protein level of p21, a cyclin-dependent kinase inhibitor. By contrast, overexpression of E2F5 dramatically increased E2F5 and CDK13 whereas decreased p21 protein level (Fig. 3c, Appendix Fig. S3B and C). Accordingly, activation of CDK13 transcription by its sgRNA increased CDK13 and E2F5 expression level and decreased p21 protein level in PC3 and 22RV1 cells. However, the effect of CDK13 overexpression was partly abrogated by depletion of E2F5 (Fig. 3d, Appendix Fig. S3D). These results indicate that there exists a positive regulation relationship between CDK13 and E2F5.

We then examined the effects of CDK13 overexpression and E2F5 knockdown either alone or together on PCa cell proliferation. MTS assay revealed that overexpression of CDK13 in PC3 and 22RV1 cells significantly promoted cell growth, but this promoting effect was abolished by transfecting cells with shE2F5 (Fig. 3e). Colony formation assay showed that both activation of CDK13 transcription and E2F5 overexpression facilitated the formation of colonies, and this effect was further enhanced by co-transfection of CDK13 sgRNA with oeE2F5 (Fig. 3f). Together, these data suggest that CDK13 and E2F5 cooperatively promote PCa cell proliferation by interacting with each other. Because both E2F5 and CDK13 are functionally related to RNA polymerase II (Pol II) [33, 34], we want to investigate whether the association of CDK13 with E2F5 is mediated by Pol II. Co-immunoprecipitation assay with Pol II antibodies followed by Western blotting indicated that the activation of CDK13 transcription strengthened the interactions between CDK13 and E2F5 or POLR2A, a subunit of Pol II, but the interaction of CDK13 with E2F5 was weakened by depletion of Pol ll by siPol II (Fig. 3g, Appendix Fig. S3E), indicating that Pol ll mediates E2F5 association with CDK13. Correspondingly, colony formation assay also revealed that CDK13 overexpression-enhanced colony formation was significantly attenuated by interfering with interactions between E2F5 and CDK13 through depleting Pol ll with siPol II in PC3 and 22RV1 cells (Fig. 3h), further supporting that Pol ll mediated-interaction of CDK13 with E2F5 facilitates the PCa cell proliferation.

### Transcriptional activation of endogenous CDK13 upregulates E2F5 expression by promoting circCDK13 formation

**Fig. 4:**
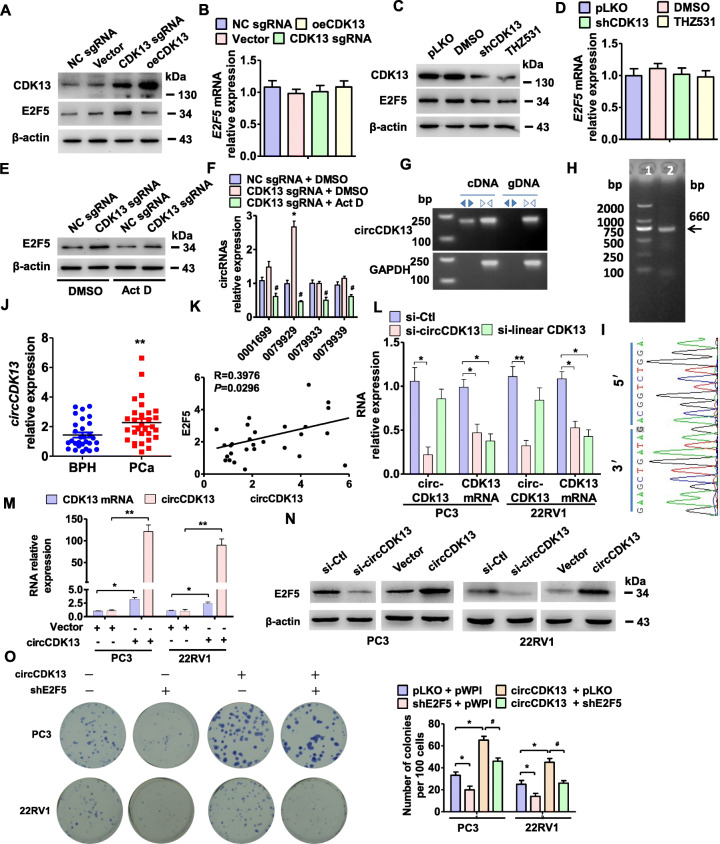
Transcriptional activation of endogenous CDK13 upregulates E2F5 expression by promoting circCDK13 formation. **a**, PC3 cells were transfected with CDK13 sgRNA, oeCDK13 or their respective control vector, and then Western blot analysis detected the CDK13 and E2F5 expression. **b**, PC3 cells were treated as in (A), RT-qPCR detected E2F5 mRNA expression. **c** and **d**, PC3 cells were transfected with shCDK13 or pLKO or treated with CDK13 inhibitor THZ531, and then Western blot (C) and RT-qPCR (D) analysis detected the CDK13 and E2F5 expression. **e** and **f**, PC3 cells were transfected with CDK13 sgRNA or NC sgRNA and treated with actinomycin D (Act D). Western blotting (E) detected E2F5 protein level, and RT-qPCR (F) detected the expression of circRNAs (hsa_circ_0001699, hsa_circ_0079929, hsa_circ_0079933 and hsa_circ_0079939). *P < 0.05 vs. NC sgRNA+DMSO; #*p*<0.05 vs. CDK13 sgRNA+DMSO. **g**, PCR was used to detect hsa_circ_0079929 (termed circCDK13) in PCa tissues by using convergent or divergent primers. Divergent primers amplify circCDK13 in cDNA but not in genomic DNA (gDNA). GAPDH was used as linear control. **h**, RT-PCR amplified full-length circCDK13 in PC3 cells, and the amplified products were confirmed by agarose gel electrophoresis. **i**, Sanger sequencing confirmed head-to-tail splicing of circCDK13. **j**, RT-qPCR detected the expression of circCDK13 in PCa and BPH tissues. **P < 0.01 vs. BPH. **k**, The correlation between circCDK13 and E2F5 mRNA expression in our clinical data was analyzed by Pearson correlation analysis (*R =* 0.3976, *P* = 0.0296). **l**, PC3 and 22RV1 cells were transfected with si-circCDK13, si-linear CDK13 or si-Ctl. RT-qPCR detected circCDK13 and CDK13 mRNA expression. Bars are mean ± SEM of triplicate samples. *P < 0.05, **P < 0.01 vs. si-Ctl. **m**, RT-qPCR examined the expression of CDK13 mRNA and circCDK13 in PC3 and 22RV1 cells transfected with circCDK13 or empty vector. *P < 0.05, **P < 0.01 vs. empty vector. **n**, Western blotting detected the E2F5 expression in PC3 and 22RV1 cells transfected with si-circCDK13, circCDK13 or their respective control. **o**, PC3 and 22RV1 cells were transfected with circCDK13 and shE2F5 either alone or together, and then colony formation assay was performed to detect the cell proliferation. *P < 0.05 vs. pLKO+pWPI, #*p*<0.05 vs. circCDK13 + pLKO

In the further experiments, we found that transcriptional activation of endogenous CDK13 by sgRNA obviously enhanced the protein expression level of E2F5, but transfection of exogenous CDK13 expressing plasmid (oeCDK13) did not affect E2F5 expression (Fig. 4a, Appendix Fig. S4A). Importantly, neither transcriptional activation of endogenous CDK13 nor transfection of oeCDK13 affected E2F5 mRNA level (Fig. 4b). Further, we used the shRNA targeting CDK13 (shCDK13) or THZ531, a selective covalent inhibitor of CDK13, to knock down CDK13 expression or to inhibit CDK13 activity in PC3 cells and confirmed that inhibition of CDK13 through these two ways did not significantly influence E2F5 mRNA and protein level (Fig. 4c and d, Appendix Fig. S4B). Additionally, we used actinomycin D to block transcription and assessed the level of E2F5 protein and found that activation of CDK13 transcription by CDK13 sgRNA in PC3 cells increased E2F5 protein level, but this inducing effect was weakened by treating CDK13 sgRNA-transfected cells with actinomycin D (Fig. 4e, Appendix Fig. S4C). These results suggest that, on the one hand, CDK13 does regulate E2F5 protein expression; on the other hand, this regulation of E2F5 by CDK13 occurs at the pre-transcriptional level.

Because some circular RNAs (circRNAs) can be transcribed together with their parental genes, and they in turn regulate the transcription of the parental gene or related genes [35], we therefore reasoned that circRNA might mediate the regulation of CDK13 and E2F5 expression. To clarify this hypothesis, we identified the highly expressed circRNAs in PC3 cells, which were formed from CDK13 gene [24]. The results of microarray analysis showed differently spliced 4 circRNAs derived from CDK13 gene [24]. Among these circRNAs, only circRNA-0079929 derived from the exon 2 of the CDK13 gene was dramatically upregulated by activation of CDK13 transcription (Fig. 4f). Therefore, we chose circRNA-0079929 to conduct all subsequent experiments and named it circCDK13. We then used divergent primers to amplify the circRNAs formed by head-to-tail splicing and confirmed that the circCDK13 was indeed observed in PC3 cells (Fig. 4g). Next, we designed a pair of additional divergent primers with partially overlapping 5′-end nucleotide bases to identify full-length circCDK13. RT-PCR, Sanger sequencing and RNase R treatment revealed that circCDK13 has a length of 660 bp and was stably expressed in PCa cells (Fig. 4h and i, Appendix Fig. S4D). Further, we measured the expression level of circCDK13 in human PCa tissues. As shown in Fig. 4j, circCDK13 expression was significantly upregulated in the PCa tissues compared with BPH tissues, and there is a significant positive correlation between circCDK13 and E2F5 mRNA level (Fig. 4k).

To further investigate whether circCDK13 is involved in the regulation of E2F5 expression, we performed loss- and gain-of-function experiments in vitro. First, we designed and synthesized the specific siRNA targeting circCDK13 (si-circCDK13) as well as targeting CDK13 mRNA (si-linear CDK13). As shown in Fig. 4l, knockdown of circCDK13 by si-circCDK13 could greatly decrease both the circCDK13 and CDK13 mRNA expression in PC3 and 22RV1 cells; whereas transfection of si-linear CDK13 did not significantly influence circCDK13 level but obviously reduced CDK13 mRNA expression. In the further experiments, we constructed a recombinant plasmid expressing circRNA (pcDNA-circCDK13) that could completely and non-redundantly express circCDK13. To confirm the circularity of circCDK13, we performed a Northern blot analysis after treatment with RNase R. The results showed that RNase R digestion reduced CDK13 mRNA level but had lesser effect on circCDK13, suggesting that circCDK13 is highly resistant to RNase R digestion (Appendix Fig. S4D and E). Also, we sequenced the circRNA expressed by pcDNA-circCDK13 by Sanger sequencing and detected the sequence at the circRNA backsplice junction (Fig. 4i, Appendix Fig. S4D and E). The results indicate that this RNA is circRNA and not linear. More importantly, transfecting PC3 and 22RV1 cells with pcDNA-circCDK13 not only increased circCDK13 level but also enhanced CDK13 mRNA expression (Fig. 4m, Appendix Fig. S4E). We next examined whether circCDK13 overexpression leads to the changes in E2F5 protein expression. As shown in Fig. 4n and Appendix Fig. S4G, knockdown of circCDK13 in PC3 and 22RV1 cells by si-circCDK13 markedly reduced E2F5 protein level, while the opposite results were observed in the two PCa cell lines overexpressing circCDK13. These findings suggest that transcriptional activation of endogenous CDK13 by sgRNA upregulates the expression level of E2F5 protein by promoting circCDK13 formation. As expected, overexpression of circCDK13 in PC3 and 22RV1 cells significantly promoted colony formation, but this promoting effect was partly reversed by depleting E2F5 with shE2F5 compared with their corresponding control (Fig. 4o). Taken together, these data indicate that circCDK13 plays its pro-proliferation effect by upregulating E2F5 expression.

### circCDK13 upregulates E2F5 protein level by sequestering miR-221-5p/449a and thus relieving their repression of E2F5 expression

**Fig. 5:**
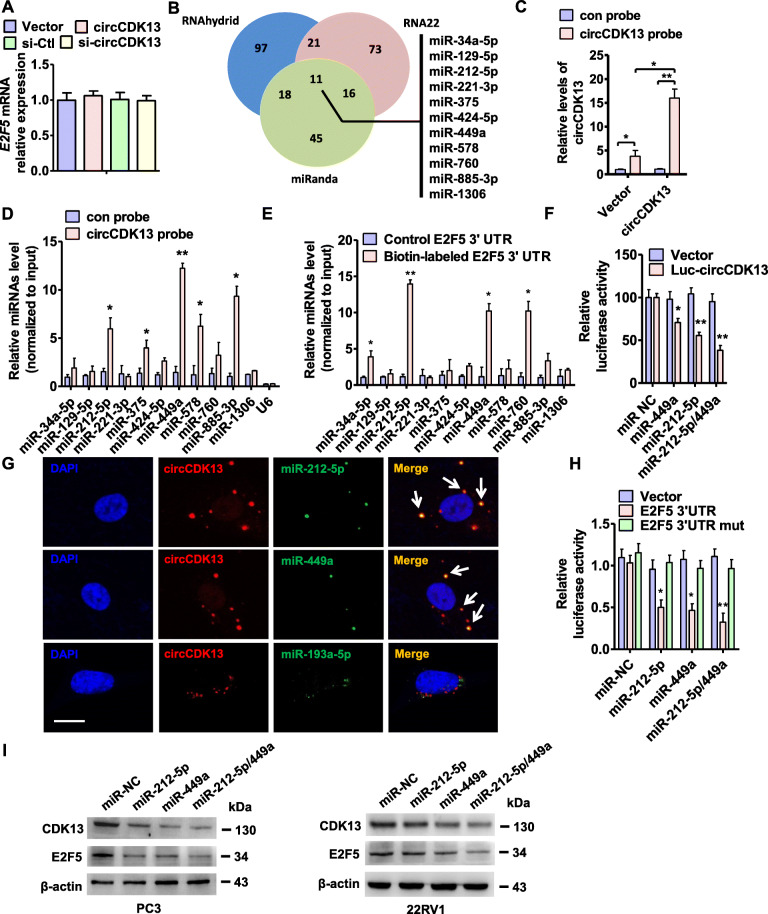
circCDK13 upregulates E2F5 protein level by sequestering miR-221-5p/449a **a**, RT-qPCR detected the expression of E2F5 mRNA in PC3 cells transfected with circCDK13, si-circCDK13 or their respective control. **b**, Venn diagram displaying potential microRNAs associated with circCDK13 sequence from three online target-prediction programs. **c**, circCDK13 or empty vector was transfected into PC3 cells, RT-qPCR detected the pulled down efficiency of circCDK13 by biotinylated probes against circCDK13. *P < 0.05, **P < 0.01 vs. con probe or empty vector. **d**, RT-qPCR detected the level of indicated miRNAs pulled down by biotinylated probes against circCDK13. *P < 0.05, **P < 0.01 vs. con probe. **e**, Biotin-labeled E2F5 3′-UTR RNA was transfected into PC3 cells followed by a biotin pull-down assay using Streptavidin-coupled Dynabeads. The miRNAs were extracted from the sedimented beads, and the relative levels of 11 candidate miRNAs were detected by RT-qPCR. *P < 0.05, **P < 0.01 vs. control E2F5 3′-UTR. **f**, PC3 cells were co-transfected with miR-449a mimic, miR-212-5p mimic or both and circCDK13-directed luciferase reporter. Luciferase activity was measured by dual-luciferase reporter assays. *P < 0.05, **P < 0.01 vs. miR-NC. **g**, RNA in situ hybridization detected the co-localization between miR-449a or miR-212-5p with circCDK13 (arrowheads) in PC3 cells co-transfected with circCDK13 expression vector and miR-449a or miR-212-5p mimic. Nuclei were counterstained with DAPI. Scale bars = 25 μm. **h**, PC3 cells were co-transfected with miR-449a, miR-212-5p or control mimic (miR-NC) and wild type or mutated (mut) E2F5 3’UTR-directed luciferase reporter. Luciferase activity was measured by dual-luciferase reporter assays. Graph bars are mean ± SEM of 3 independent experiments. *P < 0.05, **P < 0.01 vs. miR-NC or E2F5 3’UTR mut. **i**, PC3 and 22RV1 cells were transfected with miR-212-5p, miR-449a mimic or both, and then Western blotting detected the CDK13 and E2F5 expression.

Because some circRNAs can regulate downstream gene expression by sequestering miRNAs [25, 36], we identified the possible binding miRNAs of circCDK13. First, RT-qPCR showed that overexpression or knockdown of circCDK13 did not influence E2F5 mRNA expression level (Fig. 5a), implying that miRNA may play a role in the regulation of E2F5 expression. Then, we predicted the miRNA-binding sites in the circCDK13 sequence by using three online target-prediction programs, miRanda, RNA22 and Rnahydrid. As shown in Fig. 5b, circCDK13 contained sequences complementary to miR-34a-5p, miR-129-5p, miR-212-5p, miR-221-3p, miR-375, miR-424-5p, miR-449a, miR-578, miR-760, miR-885-3p, and miR-1306. We then pulled down circCDK13 by using a specific probe for circCDK13 and confirmed that circCDK13 was obviously recruited by the circCDK13 probe in circCDK13-transfected PC3 cells (Fig. 5c, Appendix Fig. S5 A and B). Next, we used RT-qPCR to detect the level of the above-mentioned 11 miRNAs in the precipitates pulled down with biotin-labeled circCDK13. As shown in Fig. 5d, miR-212-5p, miR-375, miR-449a, miR-578 and miR-885-3p were significantly enriched in the circCDK13-probe precipitates. Moreover, when circCDK13, miR-212-5p and miR-449a were at endogenous levels, the enrichment of miR-212-5p and miR-449a by circCDK13 probe has 2.9- and 2.2-fold increase, respectively, compared with control probe (Appendix Fig. S5C), suggesting that circCDK13 may function as a sponge to bind miR-212-5p and miR-449a in cells when they are at physiological levels, and thus sponge effects of circCDK13 on miR-212-5p and miR-449a are physiologically relevant. Further, we synthesized an UTP-biotin-labeled 3’UTR sequence of E2F5 mRNA and used it to pull down the miRNAs that may bind with E2F5 3’UTR. RT-qPCR showed that only miR-212-5p, miR-449a and miR-760 could be detected in the precipitates (Fig. 5e). Next, luciferase assay showed that co-transfection of miR-212-5p or miR-449a mimic with circCDK13-directed luciferase reporter significantly decreased the luciferase activity, and this inhibitory effect was further strengthened by combination of miR-212-5p with miR-449a (Fig. 5f, Appendix Fig. S5E and F). These observations suggest that both miR-212-5p and miR-449a can be sponged by circCDK13. Further, in situ hybridization also revealed that endogenous circCDK13 and miR-212-5p/miR-449a were co-localized in the cytoplasm of PC3 cells and clinical tissues (Fig. 5g, Appendix Fig. S6A and B).

Next, we investigated the function of miR-212-5p/miR-449a in the regulation of E2F5 expression. Wild type and mutated E2F5 3’UTR (E2F5 3’UTR mut) were inserted downstream of the luciferase reporter. The miR-212-5p or miR-449a mimic was then co-transfected with the luciferase reporters into PC3 cells. Compared with the control miRNA (miR-NC), miR-212-5p and miR-449a mimic was able to reduce markedly the luciferase reporter activities, but they had no significant effect on luciferase activity in the mutated binding sites of miR-212-5p and miR-449a (Fig. 5h), suggesting that both miR-212-5p and miR-449a can directly bind to E2F5 3’UTR. Accordingly, Western blot analysis also revealed that transfection of miR-212-5p or miR-449a mimic reduced CDK13 and E2F5 protein level in two PCa cell lines (Fig. 5i, Appendix Fig. S6C). These results suggest that circCDK13 upregulates E2F5 protein level by sequestering miR-221-5p/miR-449a and thus relieving miR-212-5p/miR-449a repression of E2F5 expression.

### E2F5 activates the transcription of CDK13 gene and positively regulates circCDK13 expression

**Fig. 6:**
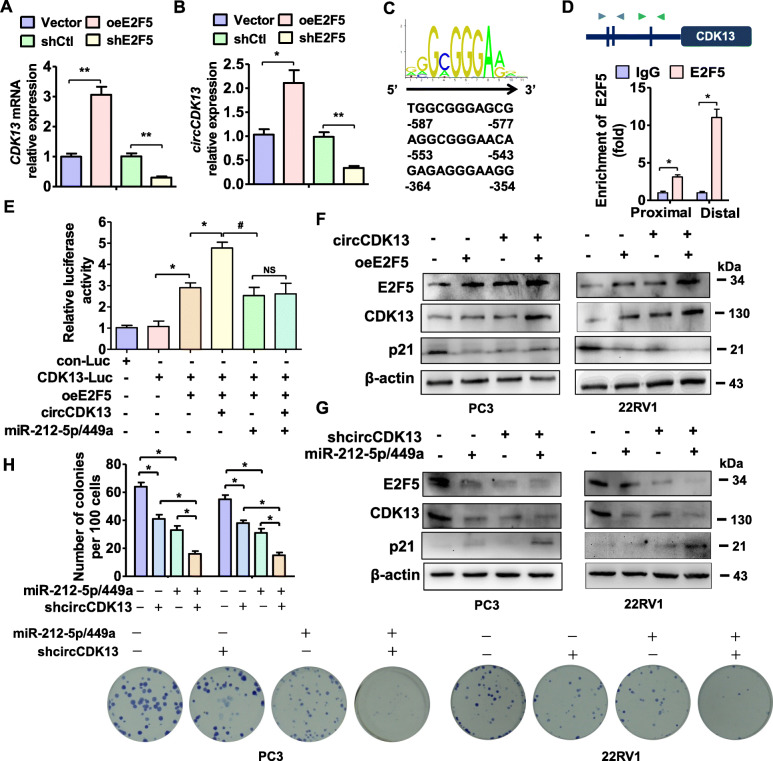
E2F5 activates the transcription of CDK13 gene and upregulates circCDK13 expression. **a** and **b**, PC3 cells were transfected with oeE2F5, shE2F5 or their corresponding control vectors, and then RT-qPCR detected CDK13 mRNA (A) or circCDK13 (B). *P < 0.05, **P < 0.01 vs. control vector. **c**, The schematic diagram shows 3 putative E2F binding sites within the 1-kb promoter region of CDK13. **d**, ChIP-qPCR detected the binding of E2F5 to the CDK13 promoter region in PC3 cells. Proximal: − 263 ~ − 459; Distal: − 444 ~ − 704. Arrowheads indicate the position of primers used for qPCR. *P < 0.05 vs. IgG. **e**, PC3 cells were transfected with the indicated constructs. Luciferase activity was measured by dual-luciferase reporter assays. *P < 0.05 vs. CDK13-Luc or CDK13-Luc + oeE2F5, #*p*<0.05 vs. oeE2F5 + circCDK13. **f** and **g**, Western blot analysis examined the E2F5, CDK13 and p21 expression in PC3 and 22RV1 cells transfected with circCDK13 and oeE2F5 either alone or together (F), or in PC3 and 22RV1 cells transfected with shcircCDK13 and miR-449a/212-5p mimic either alone or together (G). **h**, PC3 and 22RV1 cells were transfected as in (G), and cell proliferation was measured by colony formation assay. Upper panel shows the quantitative analysis of colony numbers from three independent experiments. *P < 0.05 vs. control constructs or shcircCDK13 and miR-449a/212-5p alone

Because the above evidence revealed a positive correlation between the E2F5 and CDK13 expression in PCa cells, we investigated whether E2F5, a transcriptional factor regulating genes required for cell proliferation throughout the cell cycle [37, 38], regulates CDK13 transcription. First, RT-qPCR showed that E2F5 overexpression in PC3 cells significantly increased, while knockdown of E2F5 decreased CDK13 and circCDK13 expression (Fig. 6a and b), suggesting that E2F5 positively regulates CDK13 and circCDK13 expression at the transcriptional level. We then used the online prediction software PROMO to predict the putative binding sites of E2F5 and found that 3 putative binding sites are present in the CDK13 promoter region (Fig. 6c). Chromatin immunoprecipitation (ChIP) with E2F5 antibodies followed by qPCR indicated that E2F5 directly bound to distal sequences harboring these three sites in the CDK13 promoter (Fig. 6d). Furthermore, luciferase activity assay revealed that co-transfecting PC3 cells with E2F5 expression plasmid and CDK13 promoter-reporter significantly increased luciferase activity compared with that transfected with empty vector (Fig. 6e). This promoting effect was further enhanced by circCDK13 overexpression, but was not affected by co-transfection of miR-212-5p/miR-449a mimic. These results suggest that E2F5 upregulates CDK13 and circCDK13 expression by activating the transcription of CDK13 gene.

**Fig. 7:**
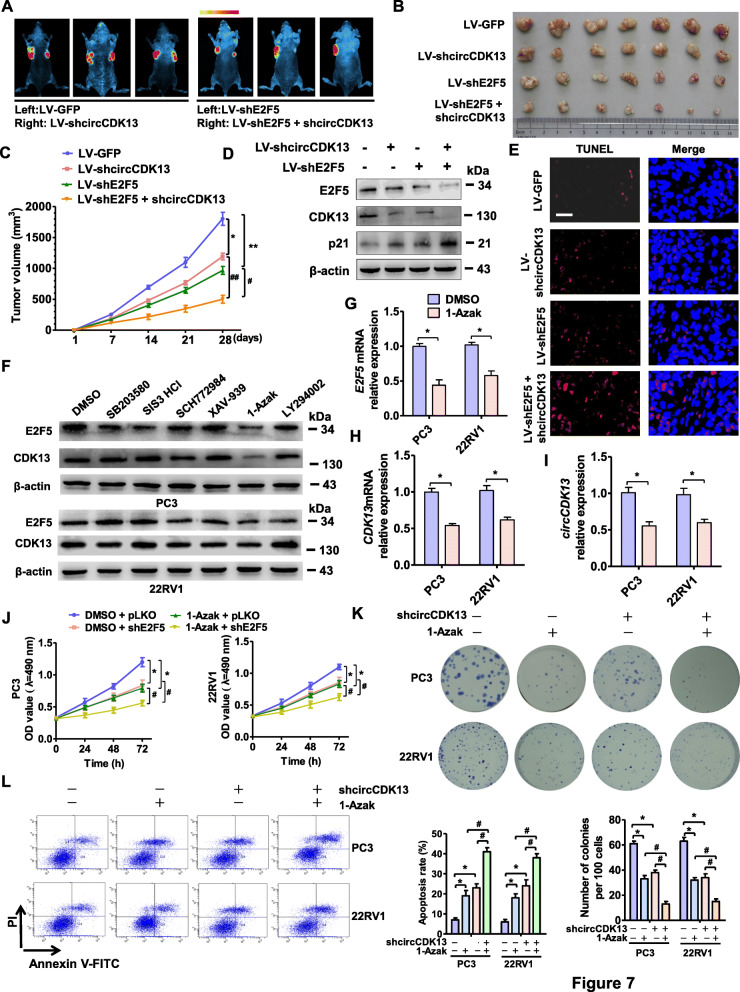
CDK13-circCDK13-miR-212-5p/miR-449a-E2F5 regulatory axis participates in prostate tumorigenesis. **a** and **b**, PC3 cells engineered to stably overexpress GFP-shcircCDK13 (LV-shcircCDK13), GFP-shE2F5 (LV-shE2F5) or negative control (LV-GFP) were injected subcutaneously in 200 μl PBS/Matrigel (50:50) into the mouse forelimb (left: LV-GFP; right: LV-shcircCDK13) (left panel), or GFP-shE2F5 (left: LV-shE2F5) or both (right: LV-shcircCDK13 + LV-shE2F5) (right panel). At the final time point (21 days after injection), the tumor volumes in each group were measured both in situ by fluorescence imaging (A) and after resection of tumors (B) (*n* = 12 in each group). **c**, Tumor volume was determined by direct measurement with calipers and calculated by the formula: volume = [(length × width2) / 2]. *P < 0.05, **P < 0.01 vs. LV-GFP (*n =* 12 in each group), #*p*<0.05, ##*p*<0.01 vs. LV-shcircCDK13 or LV-shE2F5 (*n* = 10 in each group). **d**, Western blotting detected the expression of E2F5, CDK13 and p21 in xenograft tumors prepared as in (A). **e**, The xenograft models of nude mice were prepared as in (A), the TUNEL staining detected cell apoptosis in xenograft tumors. Blue staining represents the nucleus, and red staining indicates TUNEL-positive cells. Bar = 25 μm. **f**, PC3 and 22RV1 cells were treated with indicated pharmacological inhibitors for different signaling pathways, and Western blot analysis detected the expression of CDK13 and E2F5 protein. **g-i**, RT-qPCR detected the E2F5 mRNA (G), CDK13 mRNA (H), and circCDK13 (I) expression in PC3 and 22RV1 cells treated with 1-Azak or DMSO. *P < 0.05 vs. the vehicle. **j**, PC3 and 22RV1 cells were transfected with shE2F5 or empty vector pLKO and treated with 1-Azak for 24 h, and then cell viability was measured by MTS assay. *P < 0.05 vs. the vehicle, #*p*<0.05 vs. shE2F5 or 1-Azak. **k**, PC3 and 22RV1 cells were transfected with shcircCDK13 alone or combined with 1-Azak treatment. Cell proliferation was measured by colony formation assay. Right panel shows the quantitative analysis of colony numbers from three independent experiments. *P < 0.05 vs. the vehicle, #*p*<0.05 vs. shcircCDK13 or 1-Azak. **l**, PC3 and 22RV1 cells were treated as in (K), cell apoptosis was detected by AnnexinV/PI flow cytometry. Right panel shows the apoptosis rate of three independent experiments. *P < 0.05 vs. the vehicle, #*p*<0.05 vs. shcircCDK13 or 1-Azak

To further validate the role of the formation of CDK13-circCDK13-miR-212-5p/miR-449a-E2F5 regulatory axis in PCa cell proliferation, we performed loss- and gain-of-function experiments in which circCDK13 and E2F5 were forcedly expressed or depleted in PC3 and 22RV1 cells. As shown in Fig. 7f, overexpression of circCDK13 or E2F5 increased E2F5 and CDK13 expression and decreased p21 protein level. This effect was further enhanced by co-transfecting PC3 and 22RV1 cells with circCDK13 and E2F5 expression vectors (Fig. 6f, Appendix Fig. S7A). Additionally, knockdown of circCDK13 by shcircCDK13 or repression of E2F5 expression by transfection of miR-212-5p/miR-449a mimic remarkably reduced E2F5 and CDK13 expression and upregulated p21 protein level (Fig. 6g, Appendix Fig. S7B). These effects could also be strengthened by co-transfection of them. Consistently, knockdown of circCDK13 or repression of E2F5 by miR-212-5p/miR-449a also significantly reduced colony formation (Fig. 6h). Altogether, these data suggest that the formation of circCDK13-miR-212-5p/miR-449a-E2F5 regulatory axis mediated by CDK13 upregulation contributes to PCa cell proliferation.

### CDK13-circCDK13-miR-212-5p/miR-449a-E2F5 regulatory axis participates in prostate tumorigenesis in vivo

To clarify the role of the CDK13-circCDK13-miR-212/miR-449a-E2F5 axis in prostate tumorigenesis, we established PCa xenograft models by implanting PC3 cells stably knocking down circCDK13, E2F5 or both into nude mice. As expected, the tumor volumes were apparently smaller in nude mice implanted with circCDK13- or E2F5-depleted PC3 cells than their corresponding control. Silencing both of them in PC3 cells further reduced tumor growth (Fig. 7a and b). Moreover, the mean tumor volumes and weight were significantly decreased by simultaneously silencing E2F5 and circCDK13 relative to each of them alone (Fig. 7c and Appendix Fig. S8A). In addition, we detected the expression of E2F5, CDK13 and p21 in the xenograft tumor tissues by Western blot analysis. The result showed that knockdown of circCDK13 or E2F5 notably decreased E2F5 and CDK13 expression and increased p21 protein level. Moreover, the expression changes in these genes were further enhanced when both E2F5 and CDK13 were silenced together (Fig. 7d, Appendix Fig. S8B). Further, TUNEL staining was used to detect the cell apoptosis in the xenograft tumors. As shown in Fig. 7e, depletion of circCDK13 or E2F5 facilitated cell apoptosis, and this effect was further enhanced by co-infection of LV-shE2F5 and LV-shcircCDK13. These data clearly suggest that dysregulation of circCDK13-miR-212/miR-449a-E2F5 axis mediated by CDK13 upregulation contributes to the tumor progress of PCa in vivo.

### 1-Azak functions as an anti-tumor agent in PCa cells by downregulating CDK13 and E2F5 expression and thus blocking the CDK13-circCDK13-miR-212/miR-449a-E2F5 regulatory axis

To inhibit the tumor progression caused by dysregulation of CDK13-circCDK13-miR-212/miR-449a-E2F5 pathway, we screened some pharmacological inhibitors for different signaling pathways, including p38 MAPK inhibitor, Smad3 inhibitor, ERK1/2 inhibitor, Wnt/β-catenin inhibitor, GSK-3β inhibitor and PI3K inhibitor, to identify potential CDK13 and E2F5 inhibitors. Among these compounds, 1-Azakenpaullone (1-Azak), a GSK-3β inhibitor, could markedly inhibit E2F5 and CDK13 protein expression in both PC3 and 22RV1 cells (Fig. 7f, Appendix Fig. S8C). Accordingly, treating PC3 and 22RV1 cells with 1-Azak also significantly decreased the expression of E2F5 and CDK13 mRNA and circCDK13 compared with DMSO (Fig. 7g-i). We next investigated the anti-tumor activity of 1-Azak in PCa cells. MTS and colony formation assay revealed that the cell growth was significantly inhibited by treating PC3 and 22RV1 cells with 1-Azak compared with DMSO control, and this inhibitory effect was further strengthened by knocking down E2F5 (Fig. 7j and k). Consistently, 1-Azak-treated PC3 and 22RV1 cells had a higher apoptosis rate than that of DMSO control (Fig. 7l). These data clearly demonstrate that 1-Azak inhibits PCa cell proliferation and induces apoptosis by downregulating CDK13 and E2F5 expression in PCa cells.

## Discussion

In the present study, we showed that CDK13 was significantly upregulated in PCa tissues, consistently with our results in the TCGA database. The upregulation of CDK13 depressed apoptosis and promoted proliferation of PCa cell lines. The CoIP-MS revealed that there exist a strong interaction of E2F5 with CDK13, which is involved in PCa cell proliferation. Interestingly, transcriptional activation of endogenous CDK13, but not the forced expression by transfecting a CDK13 expression plasmid into cells, remarkably promoted E2F5 protein expression by facilitating circCDK13 formation. The increased circCDK13 functions as a ceRNA of miR-221-5p and miR-449a, both of which target E2F5 3′-UTR, and thus relieves miR-221-5p and miR-449a repression of the expression of E2F5, leading to E2F5 upregulation. Subsequently, E2F5 functions as the transcriptional activator of CDK13 gene and positively regulates circCDK13 expression. To provide supporting evidence that circCDk13 can act as the ceRNA of miR-212-5p and miR-449a, we quantified the endogenous levels of circCDK13, miR-212-5p and miR-449a, and found that the expression level of circCDK13 is 26.59 times in PC3 cells and 13.49 times in 22RV1 cells over that of miR-212-5p, as well as 10.21 times in PC3 cells and 8.83 times in 22RV1 cells over that of miR-449a (Appendix Fig. S9). Moreover, there are two miR-212-5p binding sites and four miR-449a binding sites in circCDK13 sequences. Collectively, these results suggest that there are sufficient circCDK13 copies that are present in PC3 and 22RV1 cells, and thus circCDk13 can function as the sponge RNA of miR-212-5p and miR-449a in these cells. Our findings provide the first evidence that CDK13 upregulation-induced formation of the feedback regulatory loop among circCDK13, miR-212-5p/miR-449a and E2F5 is responsible for the progression of PCa. Importantly, interference of E2F5/CDK13/circCDK13/miR-212-5p/miR-449a pathway by a pharmacological inhibitor 1-Azak may be a novel therapeutic strategy for PCa.

Accumulating evidence reveals that circRNAs are not the by-products of mis-splicing or splicing errors, and a lot of circRNAs have been indicated to play an important role in cancer development, such as in prostate cancer, bladder cancer, esophageal squamous cell carcinoma and basal cell carcinoma [39]. Despite the recent advances regarding disease-related circRNAs, little is known about the biogenesis of circRNAs and the underlying molecular mechanism of circRNA-mediated gene regulation in PCa development. Our previous study found that the RNA binding protein RBM25 interacted directly with circAMOTL1L and induced its biogenesis, whereas p53 regulated epithelial–mesenchymal transition (EMT) via direct activation of RBM25 gene [24]. The neuregulin-1 intracellular domain (Nrg-1-ICD) induced circACTA2 formation in vascular smooth muscle cells through recruiting the zinc-finger transcription factor IKZF1 to the first intron of smooth muscle α-actin gene [25]. In addition, circular RNAs may be generated along with the transcription of its parental gene, and in turn regulate the expression of its parental gene [35]. Therefore, the pivotal role of circRNAs in the regulation of gene expression cannot be ignored, especially those of the circRNAs whose upregulation is accompanied by a corresponding increase in linear mRNA expression. In this study, we found that E2F5 directly bound to the CDK13 promoter and upregulated CDK13 expression and circCDK13 biogenesis. In turn, upregulation of circCDK13, as a feedback mechanism, enhanced the expression of its parental gene CDK13 via the miR-212-5p/miR-449a-E2F5 regulatory axis. It is worth noting that the positive feedback loop formed by circRNAs is often overlooked in terms of the drug resistances. For example, THZ531, an inhibitor of CDK13, potently inhibits CDK13 by irreversibly targeting a cysteine located outside the kinase domain and thus suppresses cell proliferation [40]. However, the circCDK13 produced together with CDK13 mRNA can still promote the proliferation by regulating the expression of the proliferation-related transcription factor E2F5. Therefore, blocking the positive feedback loop between circCDK13 and E2F5 in the regulation of gene expression may be one of the effective ways to prevent drug resistance.

CDK13 (also known as CDC2L5, CHED) belongs to the member of cyclin-dependent serine/threonine protein kinase family [41]. Previous studies have shown that knocking out CDK13 leads to abnormal expression of several genes involved in a variety of biological processes. Certain downregulated genes are robustly associated with transmembrane receptor protein kinase signaling, enzyme-linked receptor protein signaling pathways, cell growth regulation, helix localization, regulation of response to external stimuli, cell size regulation, and cell projection [17]. Notably, CDK13 is a crucial regulator of cell cycle progression in eukaryotes [41]. Five mammalian CDKs have been reported to be transcription-associated kinases that, together with their corresponding cyclin subunits such as CDK7/cyclin-H, CDK8/cyclin-C, CDK9/cyclin-T1 or -T2, CDK12/cyclin-K and CDK13/cyclin-K, regulate cell cycle progression and transcription [42]. A recent study of the structural and functional analysis of the CDK13/cyclin-K complex revealed that CDK13 contains a C-terminal extensional helix, a specific feature of transcriptional elongation kinases [33]. Although these complexes are related to transcription, especially, both CDK12 and CDK13 knockdown affects the expression of genes involved in RNA processing, CDK13-regulated gene sets are not affected by CDK9 or CDK12 knockdown, further suggesting that these CDK functions do not overlap with each other [41]. Moreover, several recent studies reported that CDK12 expression is dysregulated in metastatic castration-resistant prostate cancer (mCRPC) samples, and CDK12 loss results in highly recurrent gains at loci of genes involved in the cell cycle and DNA replication [18–20]. However, much less is known regarding CDK13 expression and function in PCa. In this study, we found that the expression of CDK13 was significantly increased in PCa tissues and TCGA database. Overexpression of CDK13 promoted, whereas depletion of CDK13 inhibited the proliferation of PCa cells in vitro. Importantly, we found that transcriptional activation of endogenous CDK13 by sgRNA, but not overexpression of CDK13 by its expression vector, substantially upregulated E2F5 expression by a way of epigenetics, which in turn enhanced PCa cell proliferation. Previous research has shown epigenetic regulation enables tumors to respond to changing environments during tumor progression and metastases and facilitates treatment resistance. However, a highly selective inhibitor of CDK13 that can disables triple-negative breast cancer cells progression and metastases [43]. In our immunochemistry staining result, we found that there is a strong CDK13 staining in the stromal tumor micro-environment. We hypothesize that the high expression of CDK13 in stromal tumor micro-environment may be beneficial to the migration of PCa cells.

E2F5 is a member of the E2F family of transcription factors which contain one or more evolutionarily conserved domains that bind target promoters and regulate their transcription [37]. These domains include a DNA-binding domain that determines the dimeric domain that interacts with the transcription factor protein, a trans-activated domain rich in acidic amino acids, and a tumor suppressor protein embedded in the trans-activation domain. This means that E2F5 functions as a activator or a depressor depending on the proteins which interact with it. Many studies have shown that E2F5 is a oncogene in cancer development, such as breast cancer [44], ovarian cancer [45], hepatocellular carcinoma [46], esophageal squamous cell carcinoma [47] and prostate cancer [48]. An increased gene copy number of E2F5 is detected in two independent cohorts of patients with breast cancer [44, 49], and there is a positive association of E2F5 amplification with a pathological basal phenotype and a worse clinical outcome [44]. Gandellini et al. reported that miR-205 exerts a tumor-suppressive effect in human prostate by counteracting epithelial-to-mesenchymal transition and reducing cell migration/invasion, in part through down-regulating ErbB3, E2F1, E2F5, ZEB2 and protein kinase Cε, and ectopical expression of miR-205 can halt PCa progression by the downregulation of E2F5 and E2F1 [48]. Inositol hexaphosphate (IP6) inhibits growth, and induces G1 arrest and apoptotic death of prostate carcinoma DU145 cells via decreasing the level of E2F4 as well as via increasing binding of E2F4 with pRb/p107 and pRb2/p130, thus modulating CDKI-CDK-cyclin and pRb-related protein-E2F complexes [50]. Using whole transcriptome sequencing analysis and validation of PCR assay, we confirmed a significantly increased expression of E2F5 in PCa tissues compared with BPH tissues. The upregulation of E2F5 resulted in the activation of CDK13 transcription and increase in circCDK13 biogenesis, which in turn sponges miR-212-5p and miR-449a and thus relieves their repression of the E2F5 expression, subsequently leading to the upregulation of E2F5 expression and PCa cell proliferation.

## Conclusion

In summary, our studies show that CDK13 upregulation in PCa cells leads to the formation of CDK13-circCDK13-miR-212-5p/miR-449a-E2F5 regulatory axis. These findings provide novel insights into the significance of circCDK13, CDK13, E2F5 and miRNAs in the pathogenesis and biological behavior of PCa. The circRNA generated along with the transcription of its parental gene regulates the expression of its parental gene and its downstream target gene via a positive feedback loop, which may be one of the important reasons for the resistance of tumor cells to molecular targeting drugs. Targeting this newly identified regulatory axis may provide therapeutic benefit against PCa progression and drug resistance.

## Supplementary Information

**Additional file 1.**

**Additional file 2.**

**Additional file 3.**

## Data Availability

Not applicable.

## Abbreviations

CDKs: Cyclin-dependent kinases
PCa: Prostate cancer
circRNAs: Circular RNAs
EMT: Epithelial-mesenchymal transition
miRNA: microRNA
CoIP: Co-immunoprecipitation
ChIP: Chromatin immunoprecipitation
CR: Polymerase chain reaction
UTR: Untranslated region
TCGA: The Cancer Genome Atlas
