# Supplementary material for: CDK13 upregulation-induced formation of the positive feedback loop among circCDK13, miR-212-5p/miR-449a and E2F5 contributes to prostate carcinogenesis
Source: J Exp Clin Cancer Res. 2021 Jan 4;40:2. doi: 10.1186/s13046-020-01814-5 (PMC7780414; doi:10.1186/s13046-020-01814-5)
Supplement: Supplementary file 1 — Additional file 1. [file 13046_2020_1814_MOESM1_ESM.docx]

**Supplementary table 1** Oligos used in the study

| **Oligo name** | **Sequence (5' - 3')** | **Purpose** | **Amplicon (bp)** |
| --- | --- | --- | --- |
| CDK13-F1: | CTAAGTCGTCCAAGGAGCCG | qRT-PCR for CDK13 mRNA. | 317 |
| CDK13-R1: | GCTGTAGGGACTAGGGGACA |  |  |
| CDK13-F2: | CCCTGAGCTACCAGGAGGAGATG |  | 227 |
| CDK13-R2: | CAGTGTCTTTATCCCTGGCTTTGT |  |  |
| E2F5-F: | TGCAGCAGACATCAGCTACAGA | qRT-PCR for E2F5 mRNA. | 128 |
| E2F5-R: | GTTGTAGTCATCTGCCGGGG |  |  |
| circCDK13-F | CCACTTACACCAAGCATAGGAGCC | RT-PCR for circCDK13. | 184 |
| circCDK13-R | TGTGCCTGCTACGAGATCTTGAATG |  |  |
| miR-34a-5p-F | GCTGGCAGTGTCTTAGCTGGTTGT | RT-PCR for miRNAs | N/A |
| miR-129-5p-F | CTTTTTGCGGTCTGGGCTTGC |  |  |
| miR-212-5p-F | GGCACCTTGGCTCTAGACTGCTTACT |  | N/A |
| miR-221-3p-F | GGAGCTACATTGTCTGCTGGGTTTC |  |  |
| miR-375-F | GGTTTGTTCGTTCGGCTCGC |  | N/A |
| miR-424-5p-F | GGCAGCAGCAATTCATGTTTTG |  |  |
| miR-449a-F | GGCCTGGCAGTGTATTGTTAGCTGG |  | N/A |
| miR-578-F | GGCCCTTCTTGTGCTCTAGGATTGT |  |  |
| miR-760-F | CGGCTCTGGGTCTGTGGGG |  | N/A |
| miR-885-3p-F | GAGGCAGCGGGGTGTAGTGG |  |  |
| miR-1306-F | GGCACGTTGGCTCTGGTGGTG |  | N/A |
| RNU6-1(U6)-F | GTGCTCGCTTCGGCAGCACATATAC |  | N/A |
| RNU6-1(U6)-R | AAAATATGGAACGCTTCACGAATTTGC |  |  |
| circCDK13-inf-F: | CATTCCCTTTCTTTCCCTCAGACGGTCTGGAAAATCCCGAAGC | Clone the overexpression vector for circCDK13 | 660 |
| circCDK13-inf-R: | CGTCTGCAGTTGATACTCACCTATCAGCTTCTTTATCTTCAGGCAGC |  |  |
| Pre-miR-449a-F | CTGTGTGTGATGAGCTGGCAGTG | RT-PCR for pre-miRNAs | 91 |
| Pre-miR-449a-R | TGTATATGCAATAAGACAGCAGTTGC |  |  |
| Pre-miR-212-F | CGGGGCACCCCGCCCGGAC | RT-PCR for pre-miRNAs | 110 |
| Pre-miR-212-R | GGCGGGGCCAGGCGTCGGT |  |  |
| circCDK13-FL-F | GGAGAGTAAATCTGCTGCTACAAAGGAG | Identify the full-long circCDK13 | 666 |
| circCDK13-FL-R | CTCTCCTTTCCAACTATGACTGCTTTC |  |  |
| Biotin-circCDK13-F: | ACGGTCTGGAAAATCCCGAAGC | Synthesis of biotin-U labeled circCDK13 | 660 |
| Biotin-circCDK13- T7-R: | GGATCCTAATACGACTCACTATAGGCTATCAGCTTCTTTATCTTCAGGCAGC |  |  |
| circCDK13-NB-T7-F | CTGCTGCTACAAAGGAGGAATCAG | cricCDK13 probe primer for northern blot | 393 |
| circCDK13-NB-T7-R | GGATCCTAATACGACTCACTATAGGCTGCTTCTGCGGCTCTTGC |  |  |
| Biotin-con-1: | CTTCACCCCAGGAACAAACTCCTTTGCATT | Oligo-pulldown NC Biotin | N/A |
| Biotin-con-2: | CAGCTGGATTATGGGTTGGTATTGGTCATA |  |  |
| Biotin-con-3: | GGCTGGACTGGCACTCATCTGCTGACTAAC |  |  |
| circCDK13-biotin-1: | GGGATTTTCCAGACCGTCTATCAGCTTCTTTATCTTC | Oligo-pulldown | N/A |
| circCDK13-biotin-2: | CTTCGGGATTTTCCAGACCGTCTATCAGCTTCTTTATC |  |  |
| GLO-circCDK13-660-F: | GCTCGCTAGCCTCGAGACGGTCTGGAAAATCCCGAAGC | Pmir-GLO-circCDK13 vector for Dual-Glo Luciferase Assay | 660 |
| GLO-circCDK13-660-R: | ATGCCTGCAGGTCGACCTATCAGCTTCTTTATCTTCAGGCAGC |  |  |
| circCDK13-activa-sgRNA-1 | CACCCAACGTCAGGGCGCGA | Activation of endogenous CDK13 transcription | N/A |
| circCDK13-activa-sgRNA-2 | CGCTGCCCGAGCCGAGAGCG |  |  |
| circCDK13-activa-sgRNA-3 | GAGTGAAGCGGCGACGAAGG |  |  |
| circCDK13-FAM: | GGGATTTTCCAGACCGTCTATCAGCTTCTTTATCTTC | FISH | N/A |
| circCDK13-cy3: | CTTCGGGATTTTCCAGACCGTCTATCAGCTTCTTTATC | FISH | N/A |
| miR-449a-FAM | GGCAGTGTATTGTTAGCTGG | FISH | N/A |
| miR-212-5p-FAM | CCTTGGCTCTAGACTGCTTACT | FISH | N/A |
| si-circCDK13-1-F | GCCAGUGCAUCACAAACAATT | For knock-down circCDK13. | N/A |
| si-circCDK13-1-R | UUGUUUGUGAUGCACUGGCTT |  |  |
| si-circCDK13-2-F | GGAGCAACAUGUAGCUUUATT |  | N/A |
| si-circCDK13-2-R | UAAAGCUACAUGUUGCUCCTT |  |  |
| si-linearCDK13-1-F | GCCAGUGCAUCACAAACAATT | For knock-down CDK13 mRNA. | N/A |
| si-linearCDK13-1-R | UUGUUUGUGAUGCACUGGCTT |  |  |
| si-linearCDK13-2-F | GCUGAAUUGAACAAGAAUATT |  | N/A |
| si-linearCDK13-2-R | UAUUCUUGUUCAAUUCAGCTT |  |  |
| siE2F5-F1 | GAUGGACGAUUCCAUUAAUTT | For knock-down E2F5 mRNA. | N/A |
| siE2F5-R1 | AUUAAUGGAAUCGUCCAUCTT |  |  |
| siE2F5-F2 | GCAGAUGACUACAACUUUATT |  | N/A |
| siE2F5-R2 | UAAAGUUGUAGUCAUCUGCTT |  |  |
| si-NC-F | UUCUCCGAACGUGUCACGUTT | si-RNA control. | N/A |
| si-NC-R | ACGUGACACGUUCGGAGAATT |  |  |
| E2F5-ChIP- proximal-F1: | GGAAAAGCCGAAAGAGCGAGG | ChIP | 197 |
| E2F5-ChIP- proximal-R1: | CAGCAGAGGTGGCGGAGCTAC |  |  |
| E2F5-ChIP- distal-F1: | AGTGGGCGGCACACGAATCC |  | 261 |
| E2F5-ChIP- distal-R1: | CTCTTTCGGCTTTTCCCCTCAGC |  |  |
