# Supplementary material for: CDK13 upregulation-induced formation of the positive feedback loop among circCDK13, miR-212-5p/miR-449a and E2F5 contributes to prostate carcinogenesis
Source: J Exp Clin Cancer Res. 2021 Jan 4;40:2. doi: 10.1186/s13046-020-01814-5 (PMC7780414; doi:10.1186/s13046-020-01814-5)
Supplement: Supplementary file 2 — Additional file 2. [file 13046_2020_1814_MOESM2_ESM.docx]

Supplemental Experimental Procedures

Microarray

PCa and BPH tissues were frozen in liquid nitrogen at the time of surgery and stored at −80 °C until used. Total RNA of two PCa and Two BPH samples were isolated and transcribed into fluorescent cRNA using a random priming method (Arraystar Super RNA Labeling Kit; Arraystar). The labeled cRNAs were purified using an RNeasy Mini Kit (Qiagen, Venlo, Netherlands). The concentration and specific activity of the labeled cRNAs (pmol Cy3/μg cRNA) were measured using a NanoDrop ND-1000 (Thermo Scientific, Waltham, MA, USA). One microgram of each labeled cRNA was fragmented by adding 5 μl of 10× Blocking Agent and 1 μl of 25× Fragmentation Buffer; after heating the mixture at 60 °C for 30 min, 25 μl of 2× Hybridization Buffer was added to dilute the labeled cRNA. Next, 50 μl of hybridization solution was dispensed into the gasket slide and assembled to the circRNA expression microarray slide. The slides were incubated for 17 h at 65°C in an Agilent hybridization oven (Agilent Technologies, Santa Clara, CA, USA). The hybridized arrays were washed, fixed, and scanned using the Agilent Scanner G2505C. Agilent Feature Extraction software (version 11.0.1.1) was used to analyze the acquired array images. Quantile normalization and subsequent data processing were performed using the GeneSpring GX v11.5.1 software package (Agilent Technologies). The raw and normalized microarray data have been deposited in the Gene Expression Omnibus database under accession number GSE154075.

Plasmid and lentivirus expression vector constructs

The circular RNA overexpression vectors were constructed as previously described [[1](#_ENREF_1)]. In brief, mature circRNA sequence was amplified with primers (Supplementary Table 1) and inserted into a circ-pcDNA3.1 vector (Chinese Patent No.: 2017102155320) with EcoNI and a PmlI enzyme site. All plasmids were constructed using restriction-enzyme digestion and one-step cloning (Vazyme, China) or recombinant methods. The 3' UTR sequences of E2F5 containing wild-type or mutant forms of the miR-212-5p and miR-449a target site were inserted into the Xho1 and Sal1 digested-pmir-GLO Dual-Luciferase miRNA Target Expression Vector (Promega, USA). 2 kb CDK13 promoter sequence was obtained by PCR with primer and inserted into the Mlu1 and Xho1 digested-pGL3-basic vector (Promega, USA). Luciferase assay was performed as described previously [[2](#_ENREF_2)]. In brief, PC3 cells were seeded into a 24-well plate, E2F5 reporter construct (wild-type or mutant) or the empty reporter vector was co-transfected with miRNAs mimic and pRL-TK, or co-transfected with mimic control and pRL-TK, or PC3 cells were co-transfected with pGL3-CDK13-luc vector or E2F5 or circCDK13 or miRNAs for 24 h. Luciferase activity was measured by Dual-Glo Luciferase Assay System (Promega, Madison, WI) with a Flash and Glow (LB955, Berthold Technologies) reader. The specific target activity was expressed as the relative activity ratio of firefly luciferase to Renilla luciferase.

Xenograft animal model

All animal studies were approved by the Institutional Animal Care and Use Committee of Hebei Medical University (approval ID: HebMU 20080026), and all efforts were made to minimize suffering. Male BALB/c nude mice at 4–6 weeks of age (18–22 g) were purchased from Vital River Laboratory Animal Technology Co., Ltd. (Beijing, china). 5×10^6^ PC3 cells stably expressing GFP or shcircCDK13-GFP or shE2F5-GFP or both shcircCDK13-GFP and shE2F5-GFP were harvested by trypsinization, resuspended in 0.2 mL PBS, and mixed with 50% Matrigel (Collaborative, USA); and then this suspension was injected subcutaneously into the both dorsal flanks of nude mice (one group with left: GFP and right:shcircCDK13-GFP; another group with left: shE2F5-GFP and right: shcircCDK13-GFP and shE2F5-GFP ). The length and width of mouse tumor were measured once a week with calipers or by the Fluorescent Imager Fuazon Fx (Vilber Lourmat). Then the following formula was used to calculate tumor volume (volume=[(length×width2)/2]). At the end of the experiment, the mice were euthanized by Carbon dioxide asphyxiation. At last, the tumor tissues were fixed in 4% formalin solution or flash-frozen in liquid nitrogen immediately, and stored at -80 °C until further use.

RNA Isolation and real time PCR

Total RNA was extracted from the tissues and cell as previously described [[2](#_ENREF_2), [3](#_ENREF_3)] with a miRNeasy Mini Kit (217004; Qiagen). RNA quality was determined using a NanoDrop 2000. For miRNA, reverse transcription and quantitative real-time (qRT)-PCR were performed using the miScript II RT Kit (218161) and miScript SYBR Green PCR Kit (Catalog no. 218073) with primers for specific miRNAs (Supplementary Table 1) and the internal control U6, according to the manufacturer's protocol. For large mRNA, cDNA was synthesized using a M-MLV First Strand Kit (Life Technologies) with random hexamer primers. mRNAs or circRNAs were subjected to qRT-PCR using the Platinum SYBR Green qPCR Super Mix UDG Kit (Invitrogen) and the ABI 7500 FAST system (Life Technologies). Relative transcript expression levels were normalized to GAPDH and calculated using the 2^−ΔΔCt^ formula. Supplementary Table 1 summarizes the primer sequences.

Western blot analysis

Western blotting was carried out using a standard protocol as previously described [[1-3](#_ENREF_1)]. The total protein from tumor tissues were extracted by homogenization, and cultured cells were lysated with lysis buffer (1% Triton X-100, 150 mM NaCl, 10 mM Tris-HCl, pH 7.4, 1 mM EDTA, 1 mM EGTA, pH 8.0, 0.2 mM Na3VO4, 0.2 mM phenylmethylsulfonyl fluoride, and 0.5% NP-40). Equal amounts of protein were separated on SDS-PAGE, and electrotransferred to a PVDF membrane (Millipore). Membranes were blocked with 5% milk in TTBS for 2 h at room temperature and incubated overnight with primary antibodies at 4 °C. Antibodies that were used are as follow: anti-CDK13 (1:1000, ab251955), anti-CDK4 (1:1000, ab199728), or anti-β-actin (1:1000, sc-47778), anti- E2F5 (1:500, ab44996), anti-p21 (1:500, ab188224), anti-Pol II (1:500, ab5095), anti-POLR2A (1:1000, ab140509). Membranes were then incubated with the HRP-conjugated secondary antibody (1:5000, Rockland) for 1 h at room temperature. The blots were treated with the Immobilon™ Western (Millipore), and detected by ECL (enhanced chemiluminescence) Fuazon Fx (Vilber Lourmat). Images were captured and processed by FusionCapt Advance Fx5 software (Vilber Lourmat). All experiments were replicated three times.

Morphometry and histology

Human PCa and BPH tissues were fixed in 10% neutral buffered formalin solution and then processed for routine embedding in paraffin. Ten consecutive 5-μm-thick sections were prepared for hematoxylin and eosin staining. The cross-section Images were acquired using a Leica microscope (Leica DM6000B, Switzerland) and digitized with LAS V.4.4 (Leica).

Fluorescence *In situ* hybridization

*In situ* hybridization was performed as described previously [[1](#_ENREF_1)]. In brief, cells were fixed in 4% paraformaldehyde for 5 min at room temperature and subsequently washed with PBS. Paraffin-embedded cross-sections (5-μm-thick) from clinical tissues were deparaffinized and rehydrated for in situ hybridization according to the miRCURY LNATM microRNA ISH Optimization Kit manual (Exiqon, Vedbæk, Denmark). Hybridization with fluorescence-labeled circCDK13 or miR-449a or miR-212-5p probes (Supplementary Table 1) in hybridization buffer (Exiqon) was performed during a 1-h incubation at 55°C in a thermoblock (Labnet, Edison, NJ, USA). After stringent washing with SSC buffer, samples were treated with DAPI (157574, MB biomedical) for nuclear counterstaining. Images were captured using a confocal microscope (DM6000 CFS; Leica) and processed using LAS AF software. Further images were acquired using a Leica DM6000B microscope and digitized using LAS V.4.4 software.

Immunofluorescence staining

Five-micrometer paraffin-embedded cross-sections of tissues were subjected to immunofluorescence staining as described previously[[3](#_ENREF_3)]. Sections were deparaffinized with xylene, rehydrated, and pre-incubated with 10% normal goat serum (710027, KPL, USA) followed by incubation with the following primary antibodies: anti-CDK13 (1:50, ab251955) and anti-E2F5 (1:50, ab44996). Sections were subsequently treated with the following secondary antibodies: fluorescein-labeled antibody to rabbit IgG (021516; KPL, USA). In each experiment, DAPI (1:5000, MB Biomedical) was used for nuclear counterstaining. Images were captured using a confocal microscope (DM6000 CFS; Leica) and processed using LAS AF software.

Target prediction

For finding the potential miRNA of circCDK13, we used the miRanda (www.microrna.org), RNAhybrid (http://bibiserv.techfak.uni-bielefeld.de/rnahybrid/submission.html) and RNA22(https://cm.jefferson.edu/rna22/Interactive/); For predicting the target gene of miRNA, we used Targetscan (http://www.targetscan.org).

Cell proliferation assays

MTS assay and colony formation assay were used to detect cell viability. For MTS assay, cells were seeded on 96-well plates and then transfected as indicated RNA or vectors for 24, 48 or 72 hours. 20 μL of MTSreagent (5 mg/mL; Promega, USA) was added into each well. After incubating for 3-4 h, we measured the absorbance at 495 nm by using a microplate reader (Thermo Fisher, USA).For colony formation assay, 100 cells/well culture cells were seeded into 6-well plates and culture for 1 week and then fixed with a glacial acetic acid/methanol solution. 0.5% crystal violet was used to stain the colonies. Colony numbers was counted under a microscope.

Proximity ligation assay

The proximity ligation assay (PLA) was performed as described previously [[3](#_ENREF_3)]. Briefly, A498 cells were seeded into 6 well chamber slides and cultured for 24 hours. Then 4% paraformaldehyde were used to fixate the sliders. anti-CDK13 and anti-E2F5 were used to stain the slides. Rabbit PLUS and Mouse MINUS Duolink in situ proximity ligation assay (PLA) kits were used to detect the interaction between the two proteins following the manufacturer’s protocols. Fluorescence was detected using a laser scanning confocal microscope.

Co-immunoprecipitation assay

Co-immunoprecipitation analysis was performed as described previously. Briefly, cultured cells were lysed by RIPA (25 mM Tris•HCl pH 7.6, 150 mM NaCl, 1% NP-40, 1% sodium deoxycholate, 0.1% SDS) and then lysates were immunoprecipitated with anti-CDK13 or anti-E2F5 or Pol II for 1 hour at 37°C. Protein A-agarose were added to the lysates for incubating overnight. Next day, Protein A-agarose-antigen-antibody complexes were collected by centrifugation at 12,000 g for 2 min at 4 °C and immunoprecipitation-HAT buffer was used to washed complexes for 5 times. Western blot was used to detect the bound proteins.

Chromatin immunoprecipitation (ChIP) assay

The chromatin immunoprecipitation (ChIP) assay was performed as described previously [24] and the manufacturer’s instructions (Sigma-Aldrich #17-295). In brief, PC3 cells were treated with 1% formaldehyde to cross-link proteins with DNA. The cross-linked chromatin was then prepared and sonicated to an average size of 400–600 bp. The samples were diluted 10-fold and then precleared with protein A-agarose/salmon sperm DNA for 30 min at 4 °C. The DNA fragments were immunoprecipitated overnight at 4 °C with anti-E2F5 or anti-IgG (as negative control) antibodies. After cross-linking reversal, E2F5 on CDK13 promotor was examined. Results were determined by qRT-PCR with the primers (Supplementary Table 1).

RNA immunoprecipitation (RIP) assays

RIP was performed as described previously [[1](#_ENREF_1)]. In brief, PC3 cells was harvested and lysed in NETN buffer (25 mM Tris-HCl pH 7.4, 150 mM NaCl, 1 mM EDTA, 1% NP-40 and 5% glycerol) and then cells were used to conduct RIP experiments using an anti-ILF3 antibody or IgG, and the Dynabeads™ Protein G Immunoprecipitation Kit (10007D, Thermo Fisher) according to the manufacturer’s instructions. Then the beads were washed three times with NETN buffer and RNA was isolated by using RNA Purification Kit (RNAeasy Mini Elute kit, QIAGEN) according to the manufacturer's protocol. The RNA fraction isolated by RIP was quantified by NanoDrop 2000 (Thermo-Fisher) and used for RT-qPCR with the following primers (Supplementary Table 1).

Luciferase assay

Luciferase assay analysis was performed as described previously [[2](#_ENREF_2), [3](#_ENREF_3)] . In brief, PC3 cells were seeded into a 24-well plate, CDK13 reporter construct or the empty reporter vector was co-transfected with pWPI-E2F5 and pRL-TK, or co-transfected with pWPI-vector and pRL-TK or miR-449a/212-5p. After 24 h of transfection, luciferase activity was measured using a Dual-Glo Luciferase Assay System (Promega, Madison, WI) with a Flash and Glow (LB955, Berthold Technologies) reader. The specific target activity was expressed as the relative activity ratio of firefly luciferase to Renilla luciferase.

Analyses of apoptosis

Cells grown in 6-well plates were transfected with pLKO or shCDK13, or shcricCDK13 or 1AzaK treatment or both. The Annexin V-FITC/PI apoptosis detection kit (BD Biosciences, USA) was used to detect cell apoptosis following the manufacturer’s instructions. The data analysis was performed using BD FACS Diva software (BD, USA).

RNA synthesis and Biotin pull-down

RNA was synthesized by *in vitro* transcription as previously described [[3](#_ENREF_3)] . PCR primers including T7 promoter sequences of E2F5 3' UTR were designed and amplified from PC3 cell line genomic DNA. Biotin-labeled RNA was synthesized by in vitro transcription using MEGAscript T7 transcription kit (Ambion, AM1334) and adding Biotin-16-UTP (Ambion, AM8452) in a 1:25 ratio with the transcription kit. miRNeasy Mini Kit (217004; Qiagen) was purified for transcribe RNA according to the manufacturer's instructions. Biotin pull-down was carried out to detected E2F5 3' UTR and microRNAs interaction as previously described In brief, cells were transfected with 4 μg biotin-labeled RNA for 24 h. And then cells were cross-linked with 1% formaldehyde in PBS, then quenched with 0.125 M glycine. The cells were resuspended in lysis buffer (50 mM Tris, pH 7.0, 10 mM EDTA, 1% SDS and 1 mM DTT, complete protease inhibitor, and 0.1 U/μl RNase inhibitor were freshly added) on ice for 10 min and were sonicated. The cell lysate was diluted in two times volume with hybridization buffer (750 mM NaCl, 1% SDS, 50 mM Tris, pH 7.0, 1 mM EDTA, 15% formamide, 1 mM DTT, protease inhibitor, and 0.1 U/μl RNase inhibitor). Streptavidin Dynabeads (Life Technologies) were blocked for 2 h at 4 °C in lysis buffer containing 1 mg/ml yeast tRNA and 1 mg/ml BSA and wash twice with 1 ml lysis buffer. 100 μl washed/blocked Dynabeads was added, and the whole mix was then rotated for 30 min at 37 °C. Beads were captured by magnets (Life Technologies) and washed five times with washing buffer (2×SSC, 0.5% SDS, and 0.1 mM DTT and PMSF). Beads were then subjected to RNA elution with buffer (50 mM Tris, pH7.0, 1% SDS).

TUNEL staining

TUNEL staining was performed to evaluate cell apoptosis as previously described. In brief, Xenograft tissues were fixed in 10% neutral buffered formalin solution and then processed for routine embedding in paraffin. Ten consecutive 5-μm-thick sections were prepared for TUNEL staining (Vazyme, A113). Fluorescence microscopy (DMI4000B, Leica) was used to count TUNEL-positive cells.

Statistical analysis

All of the data are presented as the means±SEM. Student's t-test was used to analysis the differences between two groups for multiple comparisons or repeated measurements, ANOVA or repeated ANOVA followed by Tukey’s post hoc test was used. P<0.05 was considered statistically significant. Statistical analysis was performed using Graphpad Prism 7 software (GraphPad Software, San Diego, CA, USA).

1. Yang Z, Qu CB, Zhang Y, Zhang WF, Wang DD, Gao CC, Ma L, Chen JS, Liu KL, Zheng B *et al*: **Dysregulation of p53-RBM25-mediated circAMOTL1L biogenesis contributes to prostate cancer progression through the circAMOTL1L-miR-193a-5p-Pcdha pathway**. *Oncogene* 2019, **38**(14):2516-2532.

2. Yang Z, Zheng B, Zhang Y, He M, Zhang XH, Ma D, Zhang RN, Wu XL, Wen JK: **miR-155-dependent regulation of mammalian sterile 20-like kinase 2 (MST2) coordinates inflammation, oxidative stress and proliferation in vascular smooth muscle cells**. *Biochimica et biophysica acta* 2015, **1852**(7):1477-1489.

3. Sun Y, Yang Z, Zheng B, Zhang XH, Zhang ML, Zhao XS, Zhao HY, Suzuki T, Wen JK: **A Novel Regulatory Mechanism of Smooth Muscle alpha-Actin Expression by NRG-1/circACTA2/miR-548f-5p Axis**. *Circulation research* 2017, **121**(6):628-635.
