# Supplementary material for: CDK13 upregulation-induced formation of the positive feedback loop among circCDK13, miR-212-5p/miR-449a and E2F5 contributes to prostate carcinogenesis
Source: J Exp Clin Cancer Res. 2021 Jan 4;40:2. doi: 10.1186/s13046-020-01814-5 (PMC7780414; doi:10.1186/s13046-020-01814-5)
Supplement: Supplementary file 3 — Additional file 3. [file 13046_2020_1814_MOESM3_ESM.docx]

**Supplemental Figures**


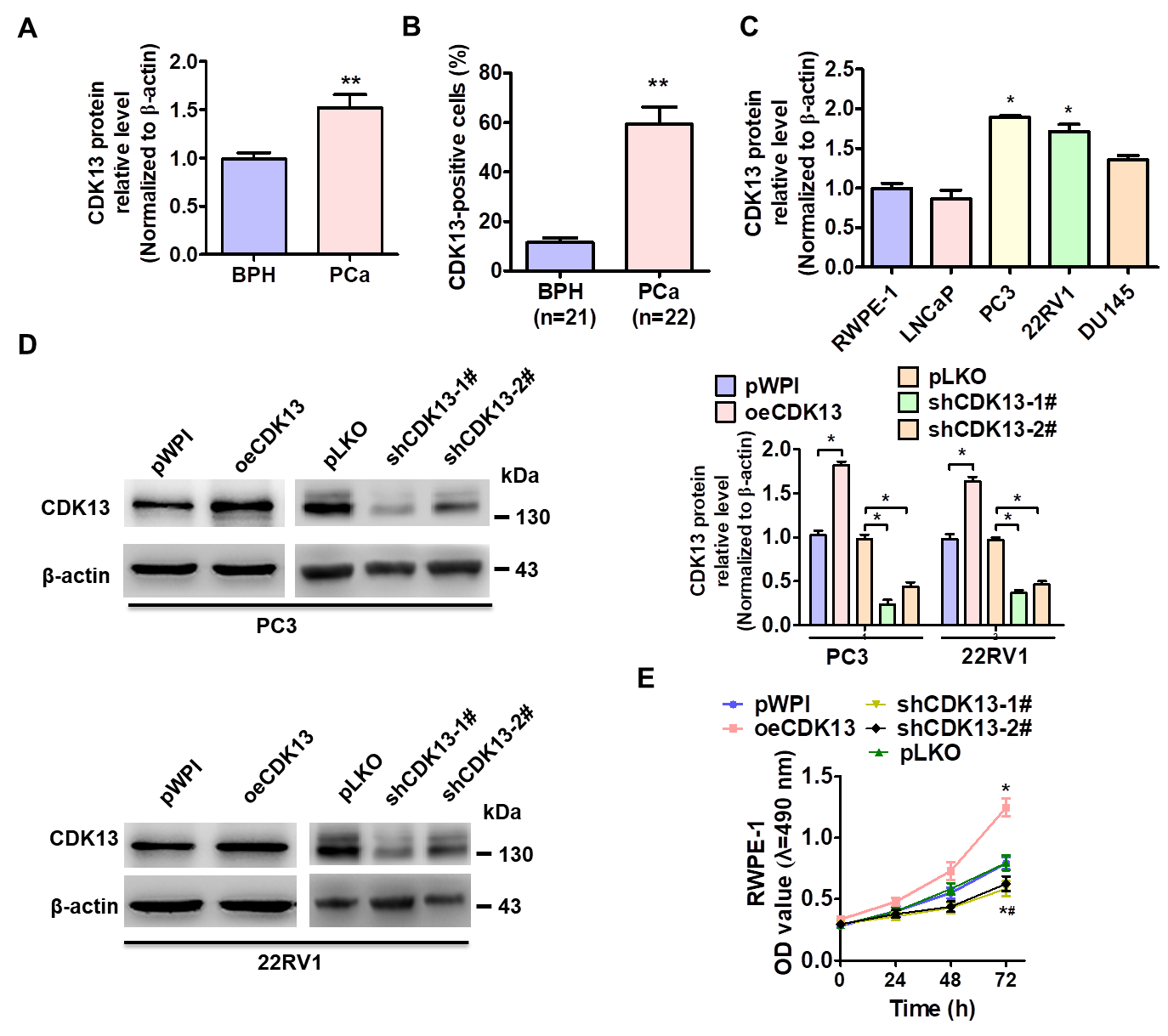


**Appendix Figure S1 Upregulation of CDK13 promotes cell proliferation.** A, Quantitative analysis of Figure 1B. Expression of CDK13 was quantitated by densitometric analysis, and values were normalized to total β–actin. n=14. *P<0.05 vs. BPH. B, Quantitative analysis of CDK13 positive cells in BPH (n = 21) and PCa (n = 22) of Figure 1D. **P<0.05 vs. BPH. C, Quantitative analysis of Figure 1F. Expression of CDK13 was quantitated by densitometric analysis, and values were normalized to total β–actin. *P<0.05 vs. RWPE-1. D, Western blot analysis detected the CDK13 expression in PC3 and 22RV1 cells transfected with oeCDK13, shCDK13-1#, shCDK13-2# or their respective control vector. The pWPI vector serves as the pWPI-CDK13 (oeCDK13) control vector, and pLKO as the pLKO-shCDK13 (shCDK13) control vector. E, Cell viability was measured by MTS assay in PC3 and 22RV1 cell lines transfected with indicated vectors. *P<0.05 vs. their respective empty vector.


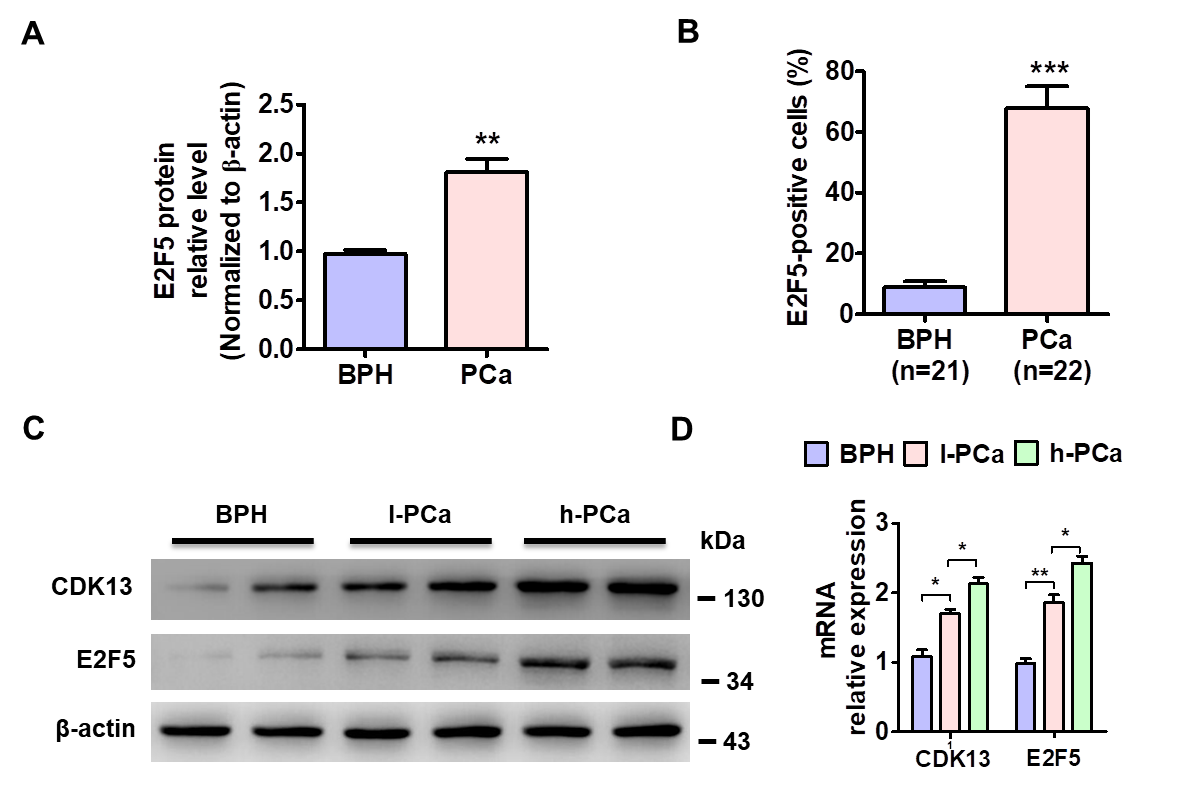


**Appendix Figure S2 A,** Quantitative analysis of Figure 2H. Expression of E2F5 was quantitated by densitometric analysis, and values were normalized to total β–actin. n=14. *P<0.05 vs. BPH. B, Quantitative analysis of E2F5 positive cells in BPH (n = 21) and PCa (n = 22) of Figure 2I. **P<0.05 vs. BPH. C and D, Western blot and quantitative real-time (qRT)-PCR analysis detected CDK13 and E2F5 expression in benign prostatic hyperplasia (BPH, n = 18), low-grade PCa (l-PCa, n = 24) and high-grade PCa (h-PCa, n = 20) . *P < 0.05, **P < 0.01 vs corresponding control.


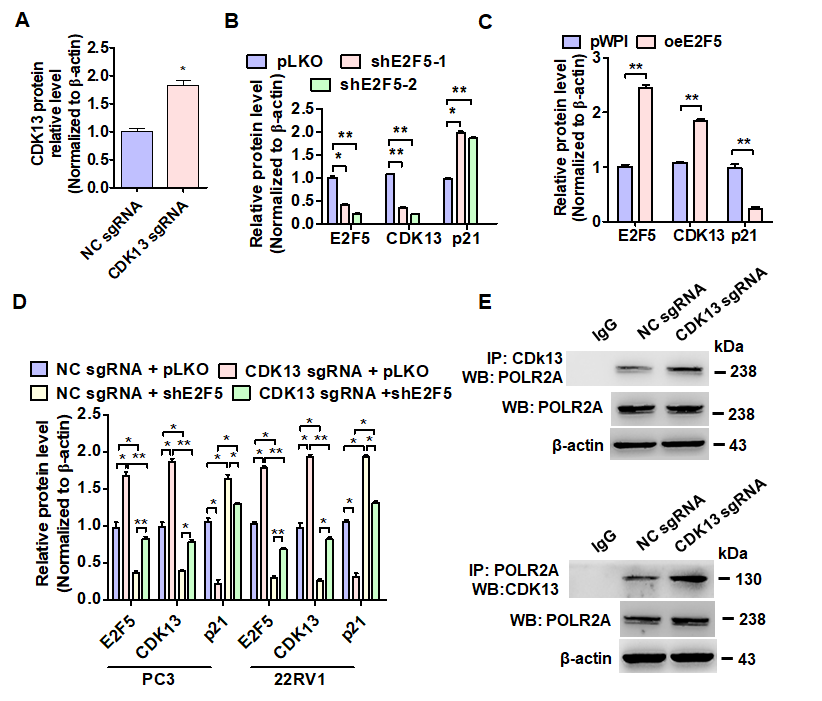


**Appendix Figure S3 CDK13 interaction with POLR2A, the subunit of Pol ll. A,** Quantitative analysis of Figure 3B. Western blot data of CDK3 quantitated by densitometric analysis, and values were normalized to total β–actin. Data are means ± SEM, n=3. *P<0.05 vs. NC sgRNA (control sgRNA). **B and C**, Quantitative analysis of Figure 3C. Expression of E2F5, CDK13 and p21 was quantitated by densitometric analysis, and values were normalized to total β–actin. Data are means ± SEM, n=3. *P<0.05, **P<0.01 vs. their corresponding control. **D**, Expression of E2F5, CDK13 and p21 was quantitated by densitometric analysis, and values were normalized to total β–actin. Data are means ± SEM, n=3. *P<0.05, **P<0.01 vs. their corresponding control. **E**, PC3 cells were transfected with CDK13 sgRNA or NC sgRNA. CoIP analysis detected the interaction between POLR2A and CDK13.


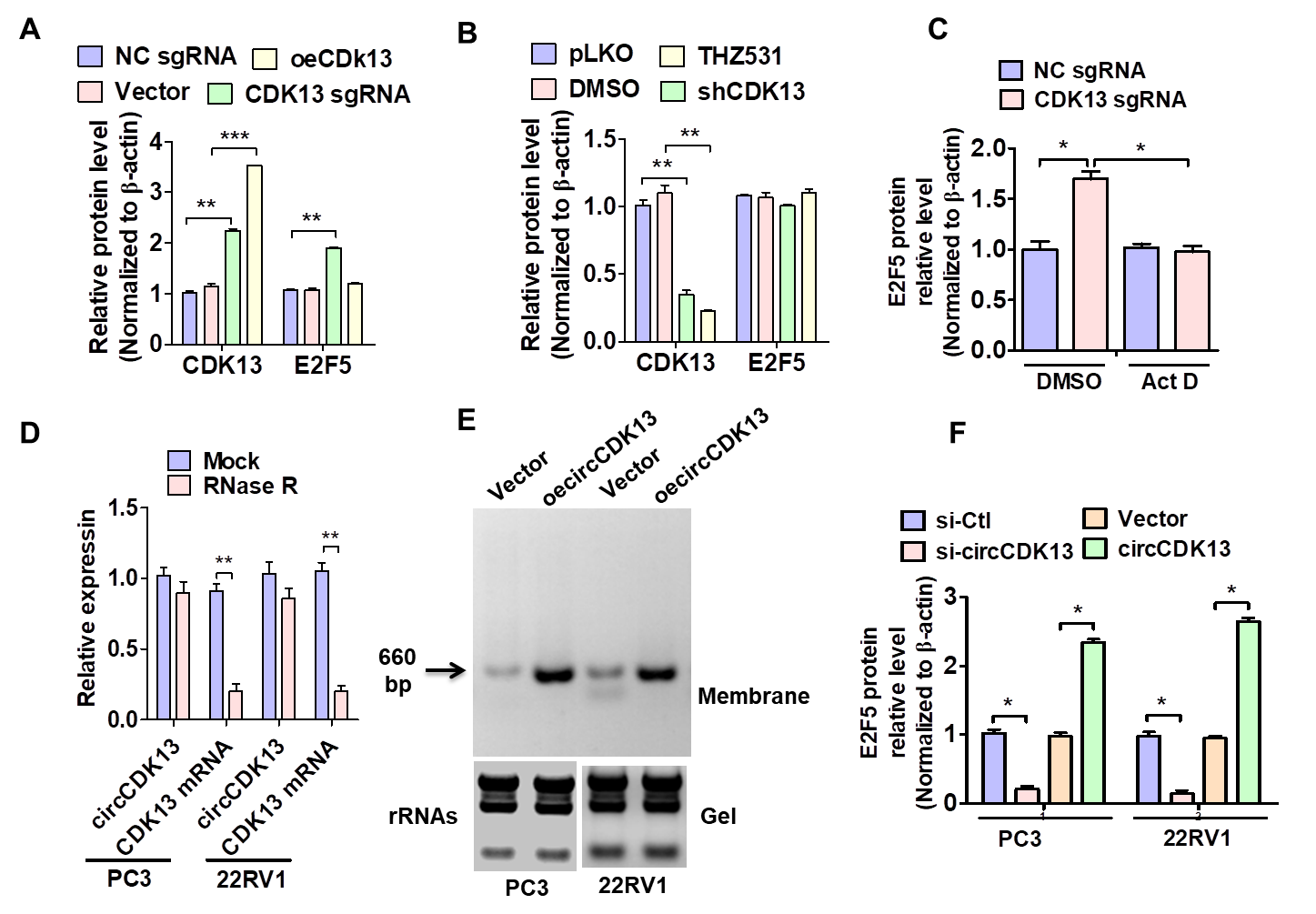


**Appendix Figure S4 Transcriptional activation of endogenous CDK13 promotes circCDK13 expression. A-C,** Quantitative analysis of Figure 4A, C and E. Western blot data of CDK13 or E2F5 quantitated by densitometric analysis, and values were normalized to total β–actin. Data are means ± SEM, n=3. *P<0.05, **P<0.01, ***P<0.001 vs. their corresponding control. **D**, The expression of circCDK13 and CDK13 mRNA in PC3 and 22RV1 cells treated with or without RNase R was detected by real-time PCR. The relative levels of circCDK13 and CDK13 mRNA were normalized to the value measured in the mock treatment. Data are means ± SEM, n=3. **P < 0.01 vs. mock. **E**, Northern blot analysis detected circCDK13 and linear CDK13 in PC3 and 22RV1 cells stably transfected with oecircCDK13 (circ-pcDNA-circCDK13) or empty vector (circ-pcDNA3.1). **F**, Quantitative analysis of E2F5 protein level of Figure 4F. *P<0.05 vs. their corresponding control.


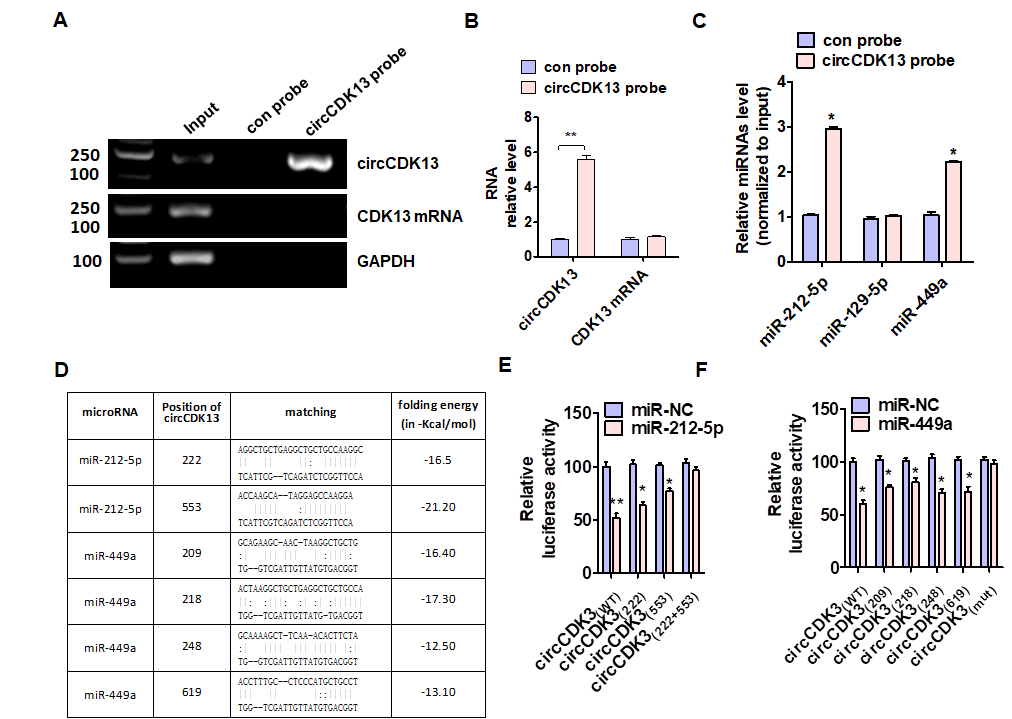


**Appendix Figure S5 circCDK13 regulates E2F5 expression by sequestering miR-221-5p/449a.** **A and B,** Lysates prepared from PC3 cells were subjected to RNA pull-down assay using circCDK13 probe and tested by RT–PCR (A) and real time PCR (B). Relative level of circCDK13 was normalized to input. GAPDH was used as negative control. Data are means ± SEM, n=3. **P < 0.01 vs. con probe. **C**, RT-qPCR detected the level of indicated miRNAs pulled down by biotinylated probes against circCDK13 in the natural condition. *P< 0.05 vs. con probe. **D**, Bioinformatics predicts the binding sites of miR-212-5p and miR-449a at circCDK13. **E and F**, PC3 cells were co-transfected with miR-212-5p (E), miR-449a (F) or control mimic (miR-NC) and wild type (WT) or mutated circCDK13-directed luciferase reporter. Luciferase activity was measured by dual-luciferase reporter assays. *P < 0.05 vs. miR-NC.


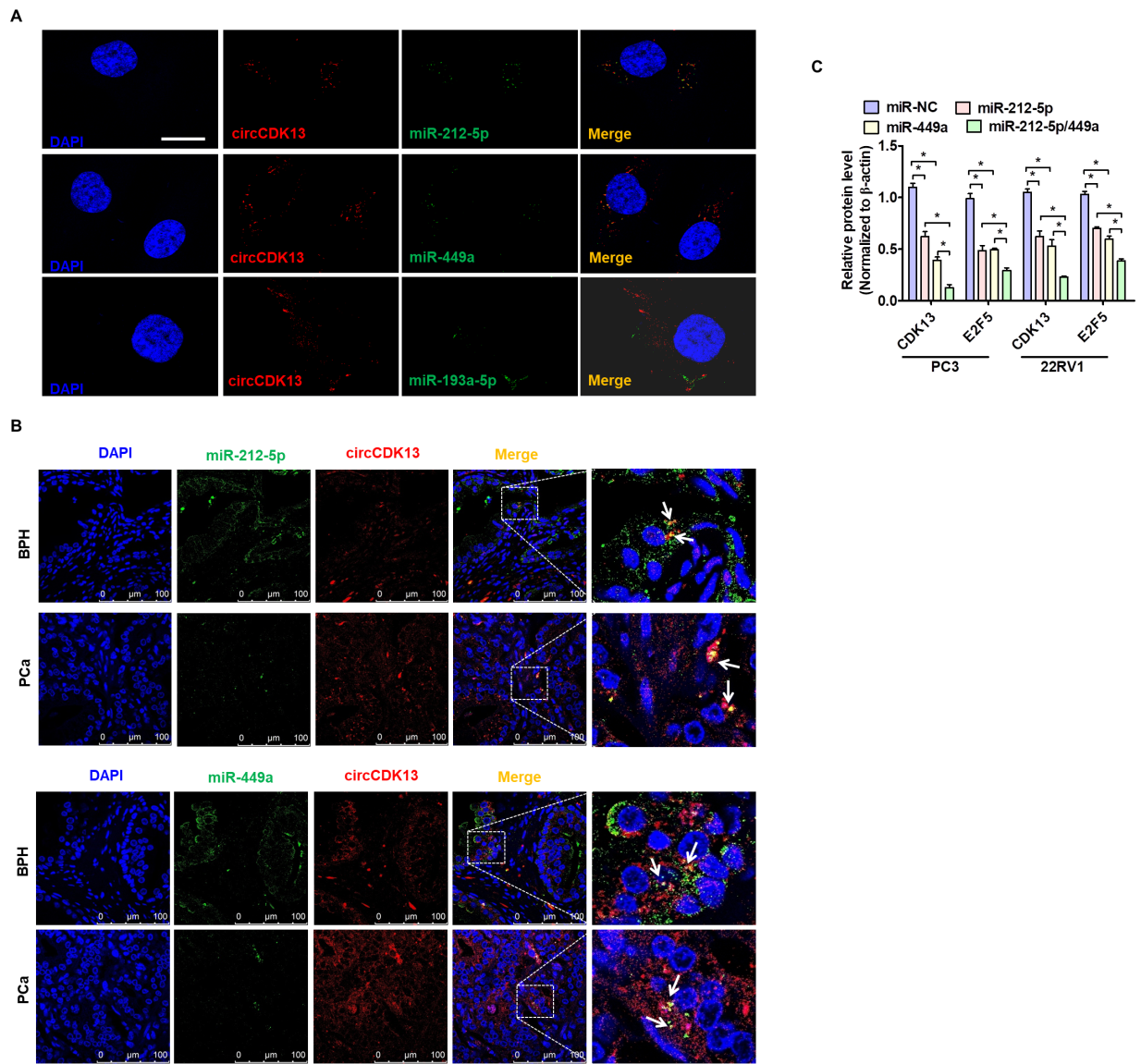


**Appendix Figure S6 circCDK13 interacts with miR-221-5p/449a.** A and B, RNA *in situ* hybridization detected the co-localization between miR-449a or miR-212-5p with circCDK13 in PC3 cells (A) or in tissues (B) under the natural condition. Nuclei were counterstained with DAPI. (A) Scale bar = 25 μm, (B) Scale bar = 100 μm. C, Quantitative analysis of Figure 5I. Expression of E2F5 and CDK13 was quantitated by densitometric analysis, and values were normalized to total β–actin. Data are means ± SEM, n=3. *P<0.05 vs. their corresponding control.


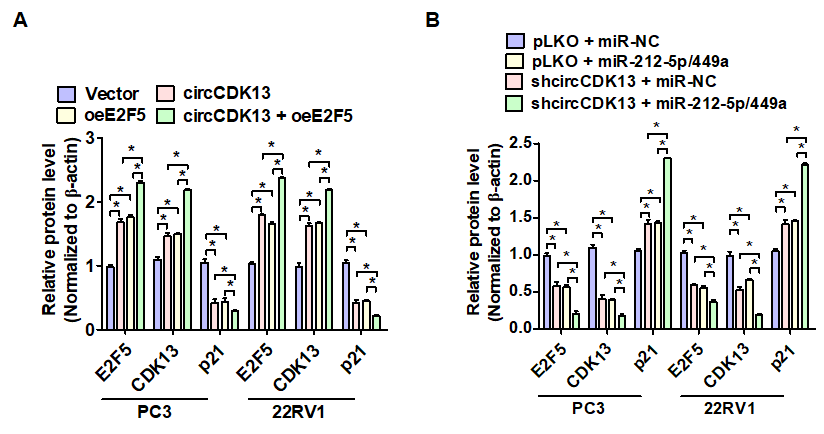


**Appendix Figure S7** A and B, Quantitative analysis of Figure 6F and G. Expression of E2F5, CDK13 and p21 was quantitated by densitometric analysis, and values were normalized to total β–actin. Data are means ± SEM, n=3. *P<0.05 vs. their corresponding control.


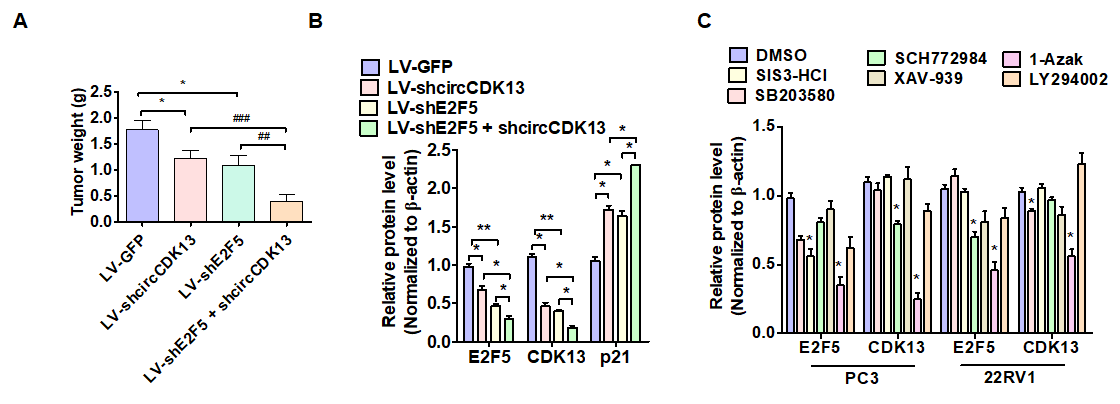


**Appendix Figure S8 A,** Quantitative analysis of tumor weight. *P < 0.05 vs. LV-GFP (n=12 in each group), ^##^p＜0.05, ^###^p＜0.01 vs. LV-shcircCDK13 or LV-shE2F5 (n = 10 in each group). **B and C,** Quantitative analysis of Figure 7D and F. Expression of E2F5, CDK13 and p21 (B) or E2F5 and CDK13 (C) was quantitated by densitometric analysis, and values were normalized to total β–actin. Data are means ± SEM, n=3. *P<0.05, **P<0.01 vs. their corresponding control.


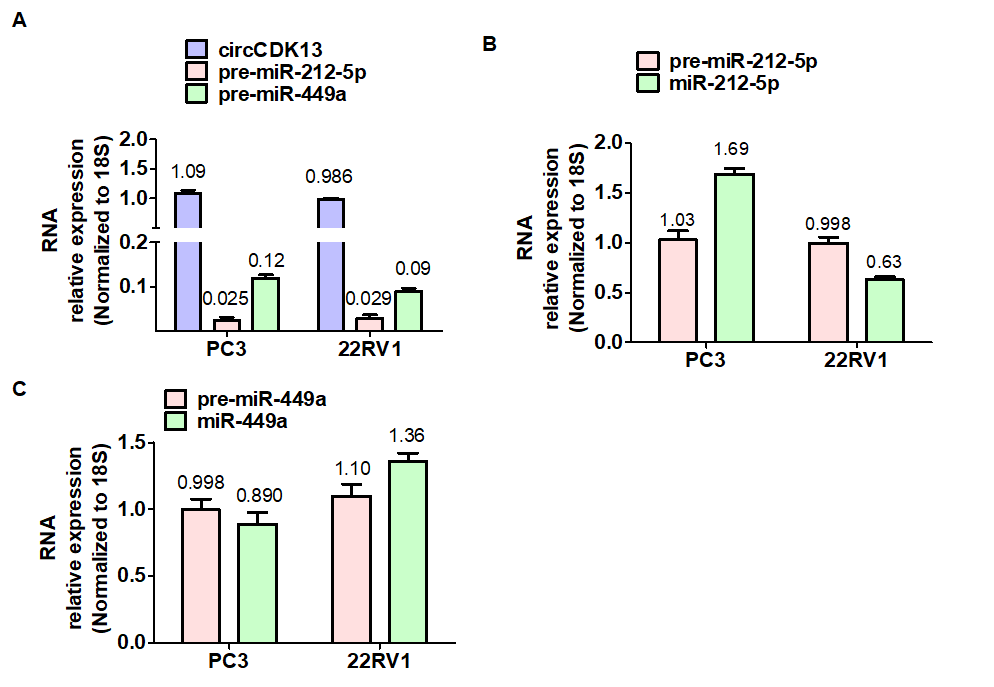


**Appendix Figure S9 Quantitative analysis of the relative expression level of circCDK13 and miRNAs. A,** First-strand cDNA was synthesized using a M-MLV First Strand Kit (Life Technologies) with random hexamer primers. Pre-miR-212-5p, pre-miR-449a or circCDK13 was subjected to RT-qPCR using the Platinum SYBR Green qPCR Super Mix UDG Kit. Relative transcript levels were normalized to 18S and calculated using the 2^−ΔΔCt^ formula. **B and C**, The mature miR-212-5p/449a and its pre-miR-212-5p/449a were quantified by using a TaqMan^TM^ probe method.
